# White matter hyperintensities and their impact in brain structure and function in alzheimer’s disease and behavioral variant frontotemporal dementia across Latin America and the United States: a cross-sectional study

**DOI:** 10.1186/s13195-025-01832-5

**Published:** 2025-08-12

**Authors:** Florencia Altschuler, Verónica Canziani, Matías Fraile-Vázquez, Raul Gonzalez-Gomez, Hernán Hernández, Sandra Baez, Joaquín Migeot, Sol Fittipaldi, Marcelo Adrian Maito, Agustina Legaz, Maria Eugenia Godoy, Sebastián Moguilner, Josephine Cruzat, Carlos Coronel-Oliveros, Enzo Tagliazucchi, Hernando Santamaria Garcia, Pablo Reyes, Diana L. Matallana, José Alberto Avila-Funes, Andrea Slachevsky, María I. Behrens, Nilton Custodio, Juan Felipe Cardona, Luis Ignacio Brusco, Martin A. Bruno, Ana L. Sosa Ortiz, Stefanie D. Pina-Escudero, Leonel T. Takada, Elisa de Paula Franca Resende, Katherine L. Possin, Maira Okada de Oliveira, Kun Hu, Brian Lawlor, Jennifer S. Yokoyama, Bruce Miller, Francisco Lopera, Adolfo Martin Garcia, Vicente Medel, Agustin Ibañez, Cecilia Gonzalez Campo

**Affiliations:** 1 Cognitive Neuroscience Center (CNC), Universidad de San Andrés, Ciudad Autónoma de Buenos Aires, Buenos Aires, 1644 Argentina; 2 Consejo Nacional de Investigaciones Científicas y Técnicas (CONICET), Ciudad Autónoma de Buenos Aires, C1033AAJ Argentina; 3 Latin American Brain Health Institute (BrainLat), Universidad Adolfo Ibañez, Metropolitan Region of Santiago, Santiago de Chile, 7910075 Chile; 4 Global Brain Health Institute (GBHI), Trinity College Dublin, Dublin, Dublin2, Ireland; 5 Universidad de los Andes, Bogotá, 111711 D.C Colombia; 6 Department of Neurology, Massachusetts General Hospital, Harvard Medical School, Boston, MA 02114 USA; 7 Instituto de Física de Buenos Aires (FIBA–CONICET), Ciudad Autónoma de Buenos Aires, Buenos Aires, 1428 Argentina; 8 Centro de Memoria y Cognición, Hospital Universitario San Ignacio, Intellectus, Bogotá, 110231 D.C Colombia; 9 PhD Program of Neuroscience, Psychiatry Department, Pontificia Universidad Javeriana, Bogotá, Colombia; 10 Mental Health Department, Hospital Universitario Fundación Santa Fe, Bogotá, Colombia; 11 Geriatrics Department, Instituto Nacional de Ciencias Médicas y Nutrición Salvador Zubirán, Ciudad de México, 14080 D.C México; 12 Geroscience Center for Brain Health and Metabolism, Santiago de Chile, Chile; 13 Memory and Neuropsychiatric Center (CMYN) Neurology Department, Hospital del Salvador & Faculty of Medicine, University of Chile, Santiago de Chile, Chile; 14 Neuropsychology and Clinical Neuroscience Laboratory (LANNEC), Physiopathology Program, Institute of Biomedical Sciences (ICBM), Neuroscience and East Neuroscience Departments, Faculty of Medicine, University of Chile, Santiago de Chile, Chile; 15 Departamento de Neurología y Psiquiatría, Clínica Alemana/Universidad del Desarrollo, Santiago de Chile, Chile; 16 Centro de Investigación Clínica Avanzada (CICA) and Departamento de Neurología y Neurocirugía, Departamento de Neurociencia, Facultad de Medicina, Hospital Clínico, Universidad de Chile, Santiago de Chile, Chile; 17 Faculty of Medicine, University of Chile, Santiago de Chile, 8380453 Chile; 18 Unit Cognitive Impairment and Dementia Prevention, Peruvian Institute of Neurosciences, Lima, 15046 Peru; 19 Facultad de Psicología, Universidad del Valle, Valle del Cauca, Cali, 760032 Colombia; 20 Departamento de Psiquiatría y Salud Mental, Facultad de Medicina, Universidad de Buenos Aires, Ciudad Autónoma de Buenos Aires, Buenos Aires, C1121ABG Argentina; 21 Instituto de Ciencias Biomédicas, Universidad Católica de Cuyo, San Juan, J5400 Argentina; 22 Instituto Nacional de Neurología y Neurocirugía, Ciudad de México, 14269 D.C México; 23 Memory and Aging Center, Department of Neurology, University of California, San Francisco, CA 94158 USA; 24 Global Brain Health Institute (GBHI), University of California, San Francisco, CA 94158 USA; 25 Grupo de Neurologia Cognitiva e do Comportamento (GNCC), Hospital das Clinicas, Faculdade de Medicina da Universidade de São Paulo, São Paulo, 05508-010 Brazil; 26 Universidade Federal de Minas Gerais, Hospital das Clínicas - EBSERH- UFMG, Belo Horizonte, MG Brazil; 27 Brigham And Women’s Hospital, Massachusetts, 02115 USA; 28 Neuroscience Research Group, Universidad de Antioquia, Medellín, 050010 Antioquia Colombia; 29 Departamento de Lingüística y Literatura, Facultad de Humanidades, Universidad de Santiago de Chile, Santiago de Chile, Chile

**Keywords:** MRI, Frontotemporal dementia, Alzheimer’s disease, White Matter Hyperintensities, Latin America

## Abstract

**Background:**

White matter hyperintensities (WMHs) are a core manifestation of normal and pathological aging and are potentially linked to geographical differences in social and physical exposomes. Previous studies have not examined the impact of WMHs burden on neurodegeneration and cognition in healthy controls (HCs) and patients with Alzheimer’s disease (AD) and behavioral variant frontotemporal dementia (bvFTD) across geographic regions. This study addressed this gap by assessing the impact of WMHs burden on participants with and without dementia from Latin America (LA) and the United States (US).

**Methods:**

The study comprised 994 participants, including HCs (*n* = 402), AD (*n* = 359), and bvFTD subjects (*n* = 233) from LA and the US. WMHs and their association with grey matter (GM) atrophy, assessed through GM volume and cortical thickness, were evaluated and compared among groups (HCs, AD, and bvFTD) in LA and the US using a voxel-wise brain imaging approach (*p* < 0.05 family-wise error-corrected for multiple comparisons, minimum cluster size = 50 voxels). Multiple regressions analysis were employed to examine geographic differences in WMHs burden, WMHs-GM associations, and the effect of WMHs on cognitive performance, as assessed by the Mini-Mental State examination.

**Results:**

In the LA cohort only, higher WMHs load was associated with greater GM atrophy across all groups (HCs, AD, bvFTD), with a specific neurodegenerative pattern involving orbitofrontal, cingulate, and temporal areas. HCs from LA showed a greater WMHs load than their US counterparts, and this effect was dependent on GM atrophy. Finally, WMHs burden negatively impacted cognitive performance in dementia subjects, with a greater effect observed in bvFTD subjects from the US.

**Conclusion:**

WMHs have a more pronounced impact on neurodegeneration across the LA cohort, with a worse impact on HCs, which also show higher WMHs burden than their US counterparts. This could increase the risk of developing dementia. Moreover, WMHs burden differentially impacts cognition, with a greater negative effect observed in bvFTD subjects from the US. These findings highlight geographic variations in WMHs-related conditions, offering valuable insights for tailored future research.

**Supplementary Information:**

The online version contains supplementary material available at 10.1186/s13195-025-01832-5.

## Background

White matter hyperintensities (WMHs) are common and nonspecific brain MRI signal abnormalities strongly associated with aging [1], cognitive decline [2], and neurodegenerative disorders [3, 4] such as Alzheimer’s disease (AD) and behavioral variant frontotemporal dementia (bvFTD). Although typically interpreted as a sign of cerebral small vessel disease (CSVD), WMHs can also be caused by neurodegeneration and neuroinflammation [5]. For example, WMHs can result from axonal damage secondary to cortical atrophy in AD [6] and in bvFTD [7], from tau and beta-amyloid deposition pathology [8] and from microglial dysfunction due to progranulin mutations [9]. Additionally, in the elderly, WMHs have been linked to less specific conditions, such as dysfunction of the glymphatic system [10].

Moreover, WMHs burden in the periventricular regions was associated with decline in general cognitive functions in healthy aging [11], while in AD and bvFTD patients, worse performance in multiple cognitive domains was associated with region-specific WMHs load [7]. WMHs burden may affect selective brain atrophy in regions associated with aging and specific dementia subtypes [12, 13]. For instance, WMHs is related to grey matter (GM) atrophy in insular and parieto-occipital regions in AD [14], in frontal regions and basal ganglia in FTD [14, 15] and with atrophy of cortical and deep brain structures in normal aging, including AD-related regions [16, 17]. Thus, WMHs can be related to specific cognitive and neural mechanisms of aging and dementia.

Aging and cognitive health are shaped by the exposome involving social and environmental factors [18], particularly affecting diverse populations like Latin Americans [19, 20]. The distinctive characteristics of this geographic region, marked by socioeconomic disadvantages [21] and cardiometabolic affections [22], may contribute to distinctive brain MRI patterns observed in normal aging and dementia. Studies across different geographic populations have uncovered differences in WMHs burden and its impact on neurodegeneration, likely tied to social and physical exposomes [23–25]. For instance, a multicenter study conducted across Europe revealed a gradient of increasing WMHs burden from north to south, contrary to the expected pattern in relation to vascular disease burden [24]. An Asian multicenter study demonstrated differential burdens of WMHs across nine cities [25]. Moreover, the impact of WMHs load on neurodegeneration was greater in non-Hispanic Black elderly individuals compared to White or Latino counterparts [12]. However, most large cohort neuroimaging studies involve white participants from developed countries. There is an urgent need to expand research by including more diverse participants. Racial and ethnic differences in the incidence and prevalence of dementia are well-documented, yet the underlying mechanisms are poorly understood. In addition, varying genetic profiles could significantly influence the development and progression of aging-related cognitive decline and dementia. Neuroimaging markers, along with their mediators and moderators, may vary among different sociocultural groups. Our aim was to narrow these knowledge gaps by studying the burden of WMHs and its impact on neurodegeneration and cognition in aging and dementia within the Latin American population. The purpose of our study was to: (i) characterize the burden of WMHs in healthy controls (HCs) and individuals with dementia (bvFTD and AD) across samples from Latin America (LA) and the United States (US), (ii) explore the association of WMHs, neurodegeneration and cognition, and (iii) investigate how the geographic region (LA) influences these associations across groups (HCs, AD, and bvFTD). We employed two analytic approaches: voxel-wise brain image analysis and multiple regression analysis. We hypothesized that the LA cohort would exhibit a greater WMHs burden compared to the US cohort, potentially leading to differing impacts on neurodegeneration and cognition.

## Methods

### Participants

The study included 994 participants (mean age = 67.68, SD = 9.04, 57.85% women), comprising 359 subjects with AD, 233 with bvFTD and 402 HCs. Participants were recruited from the Multi-Partner Consortium to Expand Dementia Research in Latin America (ReDLat) [26], spanning 10 sites in 7 countries (Argentina, Brazil, Chile, Colombia, Mexico, Peru and the United States of America). All participants underwent comprehensive neurological, neuropsychological, and neuropsychiatric assessments as well as brain MRI scans. Individuals with AD fulfilled the National Institute of Neurological and Communicative Disorders and Stroke-Alzheimer’s Disease and Related Disorders Association (NINCDS-ARDA) criteria [27], while individuals with bvFTD fulfilled the revised Rascovsky criteria [28]. Clinical diagnosis of dementia was further supported by the characteristic atrophy patterns (Fig. S1, Table S1 and Table S2). HCs exhibited preserved cognition, and had no history of neurological or psychiatric conditions. Cognition was evaluated using the Mini-Mental State Examination (MMSE) [29], which evaluates attention and orientation, memory, language, calculation, and visuospatial skills, with a maximum score of 30 indicating better performance. The study was approved by the Institutional Review Boards of each recruitment site and the Executive Committee of the ReDLat consortium. All participants signed informed consent in accordance with the Declaration of Helsinki [30].

### Neuroimaging acquisition, preprocessing and analysis

This section is reported following recommendations from the Organization for Human Brain Mapping [31]. Whole-brain structural 3DT1-weighted and FLAIR sequences were acquired for all participants across acquisition centers. Detailed scanning protocols followed by each center are detailed in Table S3. Structural T1-weighted images were preprocessed with the Computational Anatomy Toolbox (CAT12; www.neuro.uni-jena.de/cat/) implemented in Statistical Parametric Mapping software (SPM 12; Wellcome Centre for Human Neuroimaging; www.fil.ion.ucl.ac.uk/spm/software/spm12/) [32] on MATLAB R2017b. Preprocessing steps included skull stripping, grey and white matter segmentation, and normalizing to a 1.5 mm structural Montreal Neurological Institute (MNI) template [33]. Spatial Gaussian kernel smoothing of 10 mm full width at half maximum (FWHM) was then applied. Total intracranial volume (TIV) was calculated by summing the raw volumes of GM, white matter, and cerebrospinal fluid. Mean cortical thickness was calculated by averaging the left and right hemispheric cortical thickness values obtained from the CAT12 preprocessing outputs for each participant.

WMHs segmentation was performed using the lesion prediction algorithm (LPA) [34] (http://www.applied-statistics.de/lst.html) implemented in the LST toolbox, version 2.0.15, for SPM. Individual FLAIR images were used to obtain lesion probability maps [35], which were visually inspected for artifacts and discarded if artifacts were present (commonly found in the choroid plexus and basal cisterns). The probability maps were thresholded using the default value of 0.1 and non linearly normalized to the standard MNI template. The normalized WMHs maps were smoothed by a 5 mm FWHM Gaussian kernel prior to performing voxel-based analyses. Total WMHs volume (in mL) was extracted from subject-level WMHs maps. Brain lesion maps were plotted using MRIcroGL.

### Statistical analysis

#### Spatial distribution of WMHs load between disease groups

Differences in WMHs spatial distribution were assessed via voxel-wise analysis in SPM12. Second-level t-test analyses were performed using normalized WMHs lesion maps to compare HCs and neurodegenerative disease groups (AD and bvFTD) for each geographic region (LA and US). Age, years of education, sex, TIV, and scanner were controlled for as independent variables. Significance was set at *p* < 0.05 family-wise error-corrected for multiple comparisons with a cluster extent threshold of 50 voxels. The Natbrainlab white matter tract atlas, provided by MRIcron, was used to identify significant WMHs cluster locations.

#### WMHs and neurodegeneration associations

To explore the association between WMHs and neurodegeneration, we employed two approaches using voxel-wise multiple regression analyses performed on SPM12. First, we used whole-brain GM maps as the dependent variable and total WMHs volume as the independent variable of interest. Second, we used WMHs probability maps as the dependent variable and mean cortical thickness as the independent variable of interest. Covariates included age, years of education, sex, and scanner. For the first analysis, TIV was also included as covariate. Significance was set at *p* < 0.05 family-wise error-corrected for multiple comparisons with a cluster extent threshold of 50 voxels. The Automated Anatomical Labeling (AAL) and NatBrainLab atlases were used to identify GM and WMHs cluster locations, respectively.

#### Multiple linear regression analysis

Multiple linear regression analyses were employed to evaluate differences in WMHs burden and its relationship with GM atrophy between groups and geographic regions (Table 2), as well as to study the effect of WMHs burden on cognition (MMSE) (Table 3). WMHs volume was normalized to TIV and log-transformed (with the addition of a small constant, 1e-10) to achieve normal distribution. The significance threshold was set at *p* < 0.05. Analyses were performed in R (version 3.5.1).

For predicting WMHs load, two sets of regressions were conducted for each diagnosis (HCs, AD and bvFTD). Model 1 included age, sex, and geographic region as predictors. Model 2 included the same predictors as Model 1, with the addition of mean cortical thickness and the interaction term between mean cortical thickness and geographic region, to evaluate their contributions and their combined effect on WMHs.

For predicting MMSE score, a regression model was built for each diagnosis (HCs, AD and bvFTD), with total WMHs volume and geographic region entered as predictors. Age and years of education were included to account for their potential confounding effects.

## Results

### Demography and cognition

Most of the demographic variables were not balanced between group conditions and geographic regions (Table 1); thus, age, sex and years of education were controlled in each analysis. AD subjects from LA were the oldest (mean age = 72.38, SD = 8.87). Educational levels were significantly lower in all groups from LA compared to the US.

**Table 1:** Demographic and cognitive information of the full sample. Continuous variables were assessed with ANOVAs and Tukey post-hoc pairwise comparisons and are presented as mean (SD). Sex was analyzed via pearson’s chi-squared (χ2) test. The asterisk (*) indicates significant differences with an alpha level of *p* < 0.05. AD: alzheimer’s disease, bvFTD: behavioral-variant frontotemporal dementia, F: females, HCs: healthy controls, LA: Latin American countries, MMSE: Mini-Mental State Examination, US: United States.

	LA			US			Post-hoc comparisons		
	HCs (*n* = *237*)	AD (*n* = *253*)	bvFTD (*n* = *149*)	HCs (*n =* 165)	AD (*n =* 106)	bvFTD (*n =* 84)	LA	US	LA vs. US
**Age**	66.67 (9.31)	72.38 (8.87)	65.34 (9.20)	68.73 (6.64)	63.88 (8.11)	63.29 (7.17)	HCs - AD: *p <* 0.001* HCs - bvFTD: *p =* 0.10 AD - bvFTD: *p <* 0.001*	HCs - AD: *p <* 0.001* HCs - bvFTD: *p <* 0.001* AD - bvFTD: *p =* 0.84	HCs LA - US: *p* = 0.034* AD LA - US *p* < 0.001* bvFTD LA - US: *p* = 0.084
**Years of formal education**	14.69 (4.38)	11.61 (4.66)	12.90 (4.67)	17.10 (2.25)	16.62 (2.07)	16.32 (2.50)	HCs - AD: *p <* 0.001* HCs - bvFTD: *p <* 0.001* AD - bvFTD: *p =* 0.018*	HCs - AD: *p =* 0.20 HCs - bvFTD: *p =* 0.028* AD - bvFTD: *p =* 0.63	HCs LA - US: *p* < 0.001* AD LA - US *p* < 0.001* bvFTD LA - US: *p* < 0.001*
**F: M**	82:155	102:151	75:74	66:99	42:64	52:32	HCs - AD: *p =* 0.2 HCs - bvFTD: *p =* 0.0031* AD - bvFTD: *p =* 0.061	HCs - AD: *p =* 0.9 HCs - bvFTD: *p =* 0.0017* AD - bvFTD: *p <* 0.0037*	HCs LA - US: *p* = 0.3 AD LA - US *p* = 0.9 bvFTD LA - US: *p* = 0.10
**MMSE**	28.76 (1.65) *n* = 157	22.24 (4.84) *n* = 193	22.02 (6.2) *n* = 100	29.4 (0.85) *n* = 150	17.79 (7.85) *n* = 97	22.31 (6.02) *n* = 80	HCs - AD: *p <* 0.0001* HCs - bvFTD: *p* < 0.0001* AD - bvFTD: *p =* 0.7	HCs - AD: *p <* 0.0001* HCs - bvFTD: *p <* 0.0001* AD - bvFTD: *p <* 0.0001*	HCs LA - US: *p* < 0.001* AD LA - US *p* = 0.8 bvFTD LA - US: *p* < 0.001*

### WMHs burden across groups and geographic regions

**Fig. 1:**
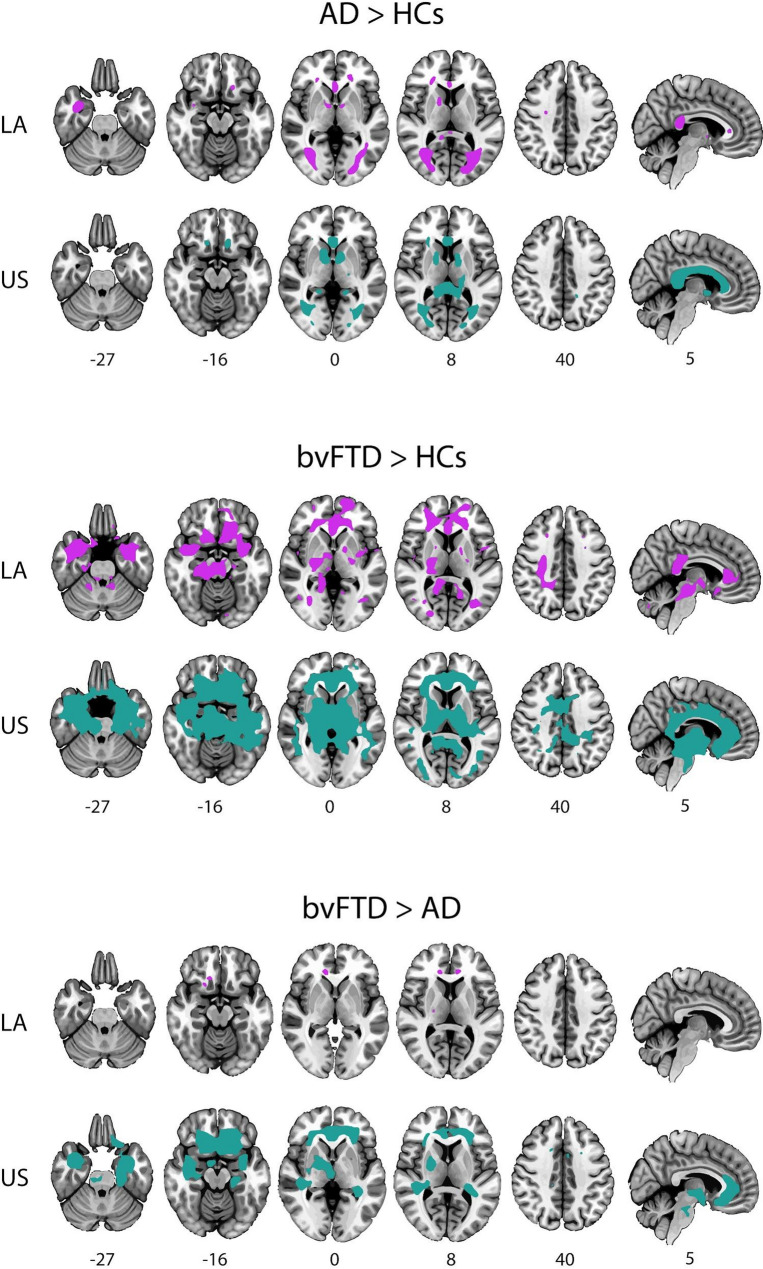
WMHs load in AD and bvFTD across LA and the US. Brain maps showing the significant areas of higher WMHs load in AD and bvFTD compared to HCs (AD > HCs and bvFTD > HCs respectively) and bvFTD compared to AD (bvFTD > AD) in LA (purple) and the US (green), controlled by age, years of education, sex, TIV and scanner. Significance was set at *p* < 0.05 FWE-corrected for multiple comparisons with a cluster extent threshold of 50 voxels. AD: Alzheimer Disease, bvFTD: behavioral variant frontotemporal dementia, FWE: family-wise error, LA: Latin America, US: United States.

Pathological groups in both geographic regions exhibited greater WMHs load than HCs as evidenced by voxel-wise analysis (Fig. 1, Table S4 and Table S5). In both regions (LA and US), bvFTD subjects displayed a predominant frontal WMHs load pattern while AD subjects exhibited a more posterior pattern. Comparison between AD and bvFTD groups, revealed higher WMHs burden in bvFTD in both LA and US regions (Fig. 1, Table S4 and Table S5). A predominant frontal pattern of involvement in bvFTD was observed in both regions (LA and US), with common tracts shared across both geographic regions and additional tracts specific to each. In LA, involved tracts included the cingulum bundle, corticospinal tract, cortico-ponto cerebellum among others (Table S4). In the US, tract-specific differences included the cingulum bundle, inferior occipitofrontal fasciculus, internal capsule, optic radiation, anterior commissure, and uncinate fasciculus among others (Table S5).

To evaluate the effect of the region (LA) on WMHs burden, we performed multiple linear regression analysis, which revealed an increased white matter lesion burden in HCs from LA (Table 2, *p* = 0.029), with no geographic differences observed in neurodegenerative groups (AD and bvFTD).

In summary, bvFTD showed a greater WMHs burden than AD in both geographic regions. HCs from LA showed an increased WMHs burden compared to their US counterparts. No differences in WMHs burden were observed between LA and the US in dementia subjects (bvFTD and AD).

**Table 2:** Multiple linear regression models to predict WMHs by group (HCs, AD, bvFTD)

	HCs					AD				bvFTD			
	Model 1			Model 2		Model 1		Model 2		Model 1		Model 2	
	Estimate (Std. error)		*p*	Estimate (Std. error)	*p*	Estimate (Std. error)	*p*	Estimate (Std. error)	*p*	Estimate (Std. error)	*p*	Estimate (Std. error)	*P*
Age	0.041 (0.005)		6.39e-16 ***	0.036 (0.005)	1.84e-13 ***	0.035 (0.005)	2.24e-11 ***	0.023 (0.005)	1.35e-06 ***	0.013 (0.008)	0.088	0.008 (0.007)	0.221
Sex [F]	0.101 (0.086)		0.240	0.167 (0.079)	0.035 *	0.106 (0.090)	0.237	0.146 (0.081)	0.070	0.106 (0.133)	0.424	0.028 (0.114)	0.807
Geographic region [LA]	0.185 (0.085)		0.029 *	2.437 (1.024)	0.018 *	0.157 (0.106)	0.139	1.152 (0.972)	0.236	0.046 (0.138)	0.741	-1.923 (1.063)	0.072
Cortical thickness				-1.180 (0.500)	0.019 *			-1.687 (0.568)	0.003 **			-3.613 (0.614)	1.42e-08 ***
Cortical thickness * geographic region [LA]				-1.456 (0.582)	0.013 *			-0.654 (0.613)	0.286			1.168 (0.708)	0.100
**Model stats**	*p* = 1.089e-14 F = 24.72 R^2^ = 0.1571			*p* < 2.2e-16 F = 33.14 R^2^ = 0.7585		*p* = 3.03e-14 F = 24.15 R^2^ = 0.1695		*p* < 2.2e-16 F = 36.34 R^2^ = 0.3398		*p* = 0.2724 F = 1.309 R^2^ = 0.01685		*p* = 6.685e-15 F = 17.83 R^2^ = 0.8594	

For each diagnostic group, two separate models were constructed: Model 1 (left columns) includes age, sex and geographic region as predictors. Model 2 (right columns) includes age, sex, geographic region, mean cortical thickness, and the interaction between cortical thickness and geographic region (Cortical thickness * geographic region). In HCs, geographic region (LA) was associated with increased lesion burden. Cortical thickness was inversely related to WMHs in all groups. The interaction between cortical thickness and region (LA) was significant in HCs, meaning that the effect of GM atrophy on WMHs burden is greater in HCs from LA. The significance threshold was set at *p* < 0.05. *Significant values. US was used as the reference group for the geographic region. AD: Alzheimer’s disease, bvFTD: behavioral variant frontotemporal dementia, F: female, HCs: healthy controls, LA: Latin American countries, US: United States. 

### WMHs and GM atrophy associations

**Fig. 2:**
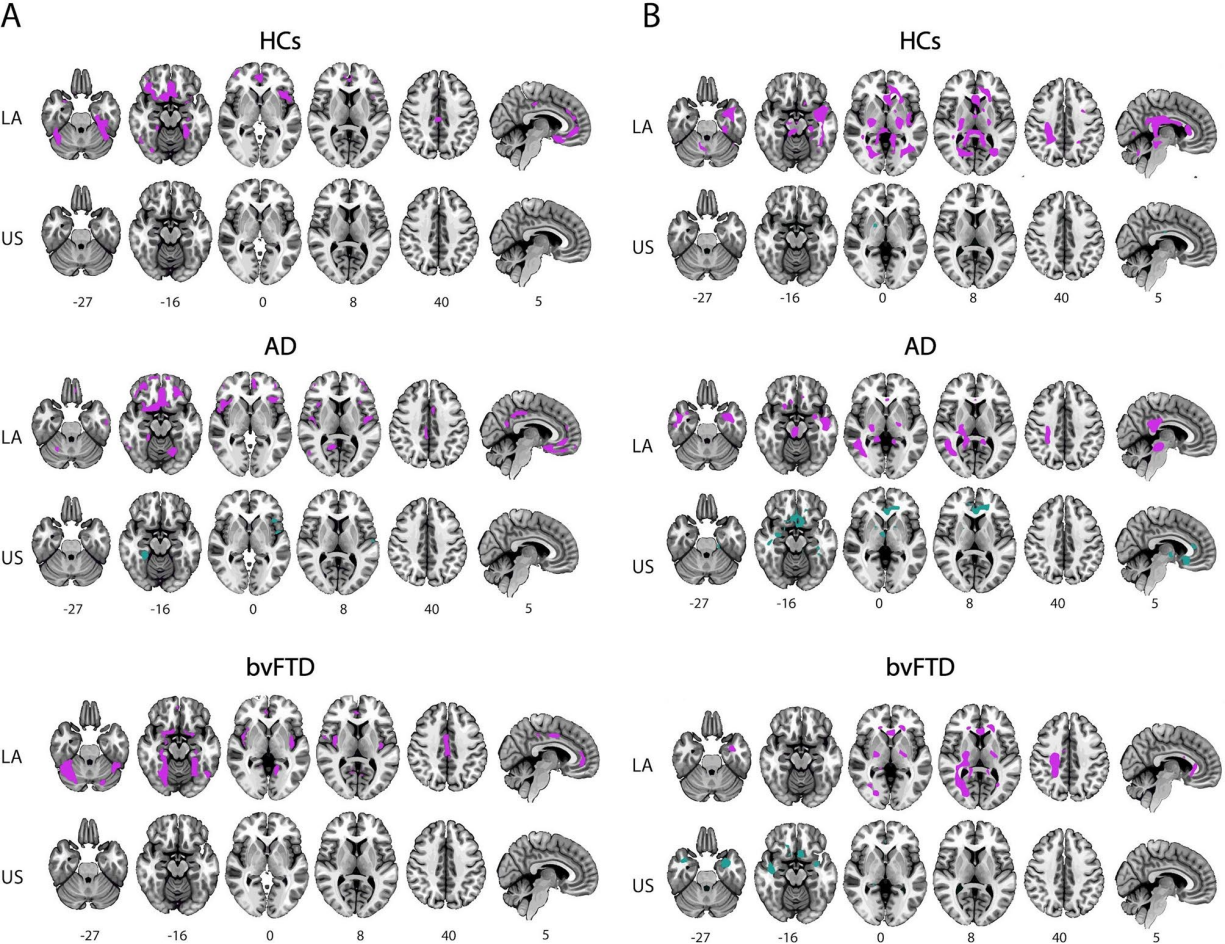
WMHs and GM atrophy associations in LA and US. **A**: Brain maps showing significant clusters of GM atrophy associated with total WMHs load in LA (purple) and in US (green) for each condition (HCs, AD, bvFTD). **B**: Brain maps showing significant tract-specific WMHs associated with cortical thinning in LA and in US regions (purple and green respectively) for each condition (HCs, AD, bvFTD). Age, years of education, sex, TIV and scanner were included as covariates of no interest. Significance was set at *p* < 0.05 FWE-corrected for multiple comparisons with a cluster extent threshold of 50 voxels. AD: Alzheimer Disease, bvFTD: behavioral variant frontotemporal dementia, FWE: family-wise error, HCs: healthy controls, LA: Latin America, US: United States

The relationship between WMHs burden and GM atrophy was assessed using a voxel-wise brain imaging approach for each group (HCs, AD, and bvFTD) and region (LA and the US). First, we employed the total WMHs volume to predict regional GM volume. Higher total WMHs volume predicted decreased GM volume in several regions across all LA groups (Fig. 2, panel A and Table S6). Notably, this association involved several of the same brain areas across all groups, including the right middle orbitofrontal cortex, the left inferior temporal gyrus, the middle cingulate cortex and fusiform gyrus bilaterally, as well as several cerebellar areas (Table S6). In AD, the neurodegenerative pattern involved large areas of fronto-temporal and parietal areas while in bvFTD involved several fronto-opercular regions, the cingulate cortex, the insula and the olfactory cortex (Table S6). The right precuneus was exclusively involved in AD, and the insula was only involved in bvFTD. Conversely, in the US, this association was only observed in AD (Fig. 2, panel A), involving several bilateral temporal regions, among others (Table S7). AD groups from both geographic regions (LA and US) showed associations in the right fusiform gyrus, in the left inferior frontal gyrus (pars triangularis) and in the right middle and inferior temporal gyrus. However, in the US, the AD group showed associations involving lesser regions with smaller clusters than in LA (Fig. 2, panel A and Table S7). Next, we investigated how mean cortical thickness could predict WMHs burden. Cortical thinning was associated with increased WMHs volume in several WM tracts across all groups (Fig. 2, panel B and Table S8), with larger clusters observed in all LA groups (HCs, AD and bvFTD) compared to the US. In LA, cortical thinning predicted WMHs burden in the corticospinal tract across all groups (HCs, AD and bvFTD), whereas in the US, no common WM tracts were implicated across groups. In HCs from LA, the affected WM tracts included the cingulum, fornix and optic radiation, among others while in the US, the cingulum and anterior commissure were implicated. In AD, the tracts involved in LA included the corpus callosum, inferior longitudinal fasciculus and uncinate fasciculus, whereas in the US, the cingulum, inferior occipitofrontal fasciculus, inferior longitudinal fasciculus, and corticospinal tract were implicated. In bvFTD, the tracts affected in LA included the corpus callosum, cingulum, fornix, and the inferior longitudinal fasciculus, while in the US, the uncinate fasciculus, cingulum, and inferior occipitofrontal fasciculus were implicated.

**Fig. 3:**
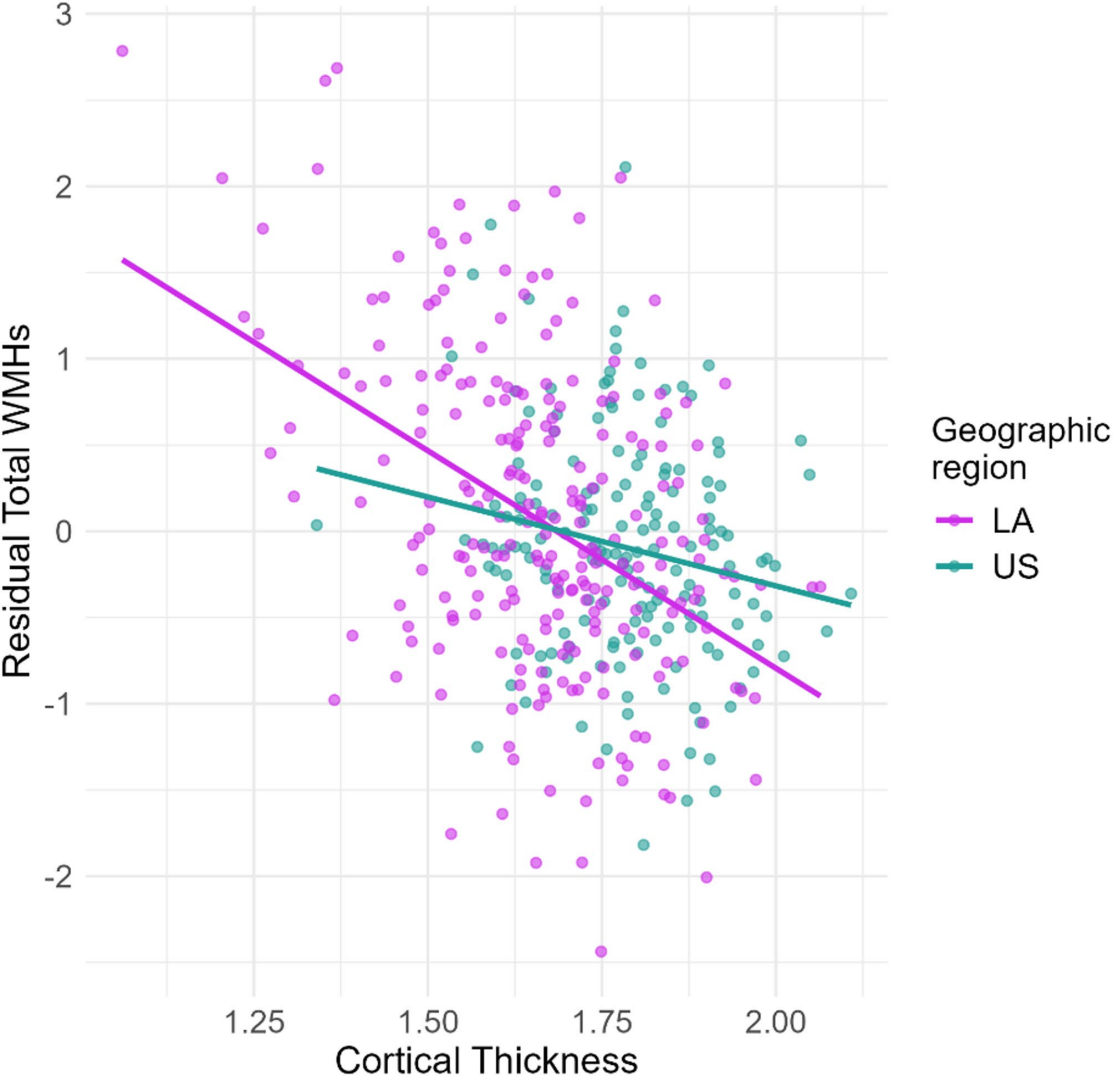
Association between cortical thickness and total WMHs by geographic region in HCs. Residuals of the total WMHs (adjusted for age and gender) are plotted against cortical thickness for each region, highlighting the regional differences across geographic groups. Lines represent linear trends within each region, and points show individual data. LA: Latin America, US: United States.

Regression models showed that cortical thinning significantly predicted WMHs burden in all groups (Table 2, HCs (*p* = 0.019), AD (*p* = 0.003) and bvFTD (*p* = 1.42e-08)). Moreover, the interaction term between cortical thickness and geographic region was significant for HCs (Table 2, *p* = 0.013), suggesting that the effect of cortical thickness on WMHs burden differs by geographic region, with a region-specific negative impact observed in HCs from LA (Fig. 3). In line with these findings, the inclusion of cortical thickness and its interaction with geographic region in Model 2 led to a consistent increase in R² values across all diagnostic groups compared to Model 1 (HCs: 0.16 to 0.76; AD: 0.17 to 0.34; bvFTD: 0.02 to 0.86) (Table 2), indicating that incorporating neurodegeneration measures improved the models’ explanatory power for WMHs burden. Overall, these results highlight a greater negative interaction between WMHs burden and neurodegeneration in the LA cohort, emphasizing regional differences in the effects of WMHs burden.

### WMHs correlates of cognitive performance

**Fig. 4:**
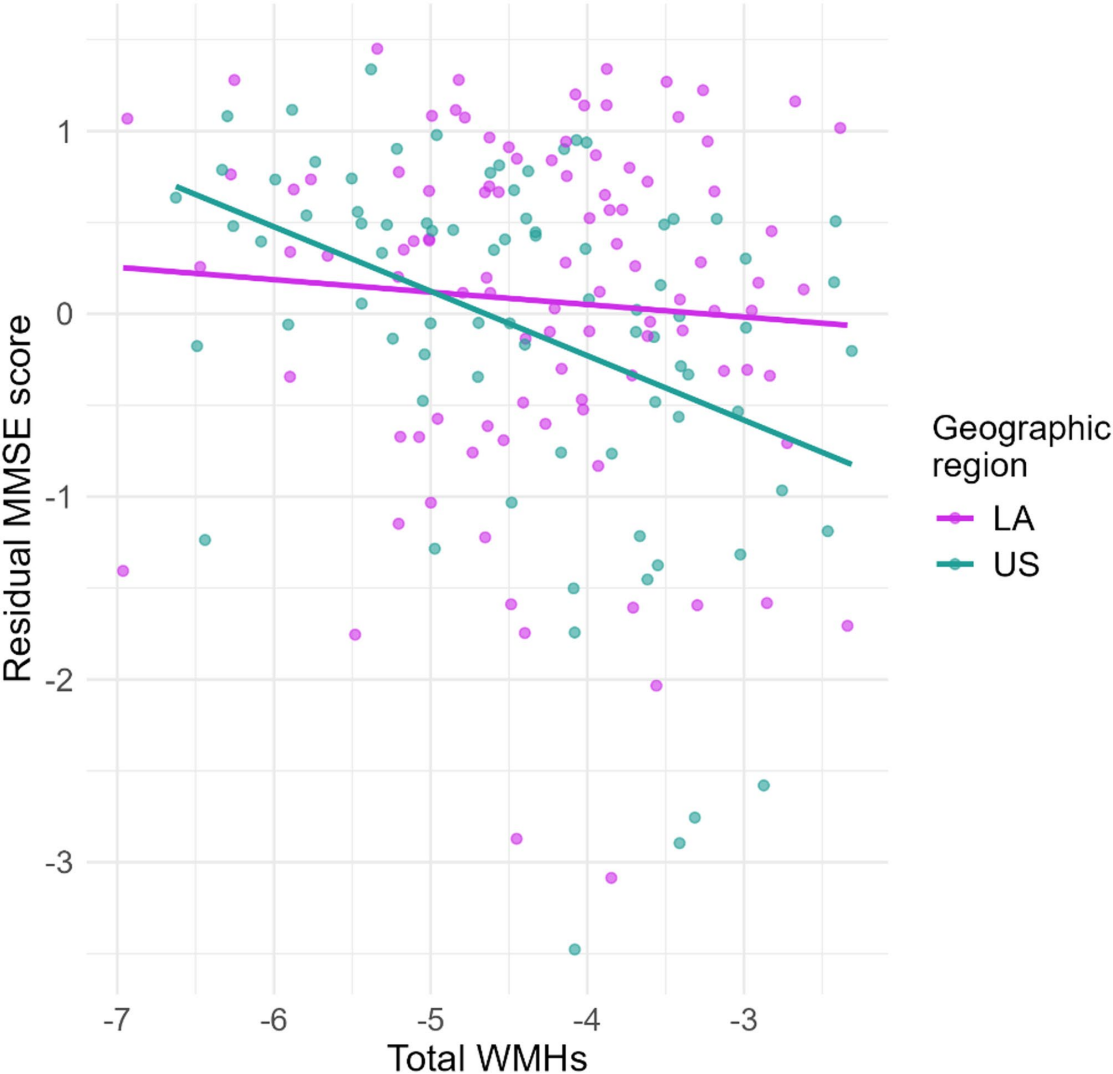
Association between total WMHs and MMSE scores by geographic region in bvFTD. Residuals of MMSE scores (adjusted for age and education) are plotted against WMHs volume for each region, highlighting the region-specific effects on cognitive outcomes across geographic groups.Lines represent linear trends within each region, and points show individual data. LA: Latin America, MMSE: Mini-Mental State Examinantion, US: United States.

Multiple linear regression models revealed that total WMHs burden contributed to explain poorer MMSE score in AD and bvFTD patients (Table 3), with stronger effect on bvFTD. The interaction between total WMHs and region (LA) was significant in bvFTD (Table 3, *p* = 0.031), indicating that the association between WMHs burden and MMSE score was weaker in the LA region compared to the US (Fig. 4). In summary, total WMHs load emerged as a key predictor of cognitive decline in dementia groups, with a stronger effect in bvFTD subjects, and in particular from the US.

**Table 3:** Multiple linear regression models to predict MMSE score

	HCs (*n* = 307)		AD (*n* = 290)		bvFTD (*n* = 180)		
	Estimate (Std. error)	*p*	Estimate (Std. error)	*p*	Estimate (Std. error)	*p*	
Age	-0.046 (0.010)	4.92e-06 ***	0.136 (0.043)	0.002 **	0.097 (0.055)		0.080
Years of education	0.061 (0.021)	0.004 **	0.411 (0.084)	1.73e-06 ***	0.465 (0.109)		3.01e-05 ***
Total WMHs	0.153 (0.142)	0.281	-1.707 (0.657)	0.010 **	-2.246 (0.596)		0.0002 ***
Geographic Region [LA]	-1.360 (0.952)	0.154	6.287 (3.931)	0.111	9.382 (3.756)		0.013 *
Total WMHs * Geographic Region [LA]	-0.165 (0.175)	0.345	0.061 (0.795)	0.939	1.818 (0.836)		0.031 *
**Model stats**	*p* = 2.137e-09 F = 10.61 R^2^ = 0.1498		*p* = 4.953e-15 F = 17.41 R^2^ = 0.2346		*p* = 1.35e-05 F = 6.534 R^2^ = 0.1581		

Multiple linear regression analyses were run to predict MMSE score from total WMHs volume and geographic region. Age and years of education were included as covariates. Total WMHs burden contributed to explain a worse performance on MMSE in AD and in bvFTD patients. The interaction between total WMHs and region (LA) was significant in bvFTD, meaning that the effect of WMHs in MMSE is weaker in LA compared to the US. The significance threshold was set at *p* < 0.05. *Significant values. AD: Alzheimer’s disease, bvFTD: behavioral variant frontotemporal dementia, HCs: Healthy controls, LA: Latin American countries, US: United States.

## Discussion

In this study, we investigated WMHs volume and its association with neurodegeneration and cognition in healthy aging and dementia in LA, contrasting our findings with a sample from the US. Our key findings are as follow: (i) In LA, associations between WMHs burden and GM atrophy were more pronounced across all groups (HCs, AD and bvFTD), (ii) HCs from LA exhibited a greater WMHs load compared to their US counterparts and this was influenced by GM atrophy, and (iii) the impact of WMHs on cognition was most pronounced in bvFTD subjects.

We observed a relationship between total WMHs burden and GM volume loss in several brain areas across all groups (HCs, AD, and bvFTD) within the LA sample, which was not present in the US cohort. Commonly implicated GM areas across all LA diagnostic groups included the orbitofrontal cortex, temporal and cingulate areas. Notably, these areas have previously been linked to WMHs burden. The presence and progression of WMHs have been associated with medial temporal atrophy progression in AD [36]. Moreover, the connectivity of the orbitofrontal cortex has been shown to vary according to WMHs burden in a non-demented population [37]. In addition, these brain regions are known to support complex cognitive and emotional processing in both normal aging [38] and dementia [39, 40].

Additionally, cortical thinning was associated with larger WMHs-affected areas in LA groups, notably involving the corticospinal tract across all diagnostic groups. This consistent pattern, observed across all diagnostic groups within the LA region, suggests a geographic origin potentially linked to regional factors such as environmental or socioeconomic influences, contributing to an additional burden in LA. Supporting this hypothesis, previous studies have shown that the structural integrity of the corticospinal tract, as measured by fractional anisotropy, is associated with socioeconomic status, including education and income levels [41].

Disease-specific patterns were also evident in the WMHs-GM associations, with the precuneus uniquely affected in AD and the insula specifically involved in bvFTD, highlighting potential additive mechanisms of regional burden in LA that may exacerbate dementia-related pathology. The precuneus is increasingly recognized as a vulnerable and early-affected region in various neurodegenerative diseases, especially AD [42, 43]. Remarkably, in Black American populations, the precuneus has been identified as an early and vulnerable brain region associated with amyloid deposition, vascular burden, and disruptions within the default mode network—factors that may collectively reflect the cumulative impact of social, vascular, and biological risk [44, 45]. These findings suggest that the precuneus may act as a hub for early AD-related changes in underrepresented populations. The precuneal involvement observed in AD subjects from LA may similarly reflect these vulnerability mechanisms, warranting further investigation.

Beyond these region- and population-specific patterns, it is crucial to understand the general mechanisms linking WMHs and neurodegeneration, a relationship for which several explanatory pathways have been proposed. On one hand, WMHs may lead to cortical thinning through secondary neurodegeneration along disrupted white matter tracts [46–48]. WMHs represent localized disruptions in white matter integrity, often due to CSVD [49]. They also serve as markers of broader cerebrovascular and metabolic dysfunction, consistent with the “tip of the iceberg” model of CSVD [50], where visible WMHs represent only the detectable portion of a global pathological process affecting both white and GM. On the other hand, neurodegeneration could contribute to the development and progression of WMHs [6]. In AD and related dementias, WMHs burden may partially result from proteinopathies (e.g., tau, amyloid) [8], leading to Wallerian degeneration [5]. These neurodegenerative and vascular processes are not mutually exclusive and likely act synergistically [51].

Regional differences in vascular risk [52, 53], genetics [54], and environmental stressors (exposomes) [18] likely contribute to the variations in both the burden of WMHs and GM atrophy, as well as their interactions. These factors may help explain the differences observed between LA and US. Supporting this, Rizvi et al. [12] reported stronger associations between WMHs burden and cortical thinning in frontal and parietal regions among vulnerable populations, particularly non-Hispanic Black individuals compared to White older adults. Further research is needed to elucidate these mechanisms and their contribution to regional disparities.

Remarkably, HCs from LA exhibited a greater WMHs load than their US counterparts, and this effect was partially mediated by cortical thinning. This structural vulnerability in normal aging could represent a risk factor for developing dementia. Future research should investigate whether genetic factors, socioeconomic conditions, educational attainment, environmental influences (e.g., diet, physical activity, stress), access to healthcare, and social engagement collectively contribute to a harmful allostatic load that exacerbates WMHs burden in LA.

Our findings also emphasize the significant role of WMHs burden in cognitive decline among dementia patients, particularly in bvFTD. Albeit the accepted negative role of WMHs in cognitive performance, the extent to which it affects cognition is still under debate. Moreover, WMHs burden is generally associated with vascular and AD pathology. Here, we show a predominant role of WMHs in cognitive decline among FTD subjects, in line with prior research [7, 55]. Future studies should examine more in detail the role of white matter and GM pathology across dementia subtypes.

Similar to the white matter-GM associations, the impact of WMHs burden on cognition exhibited regional differences, with a more pronounced effect in bvFTD subjects from the US compared to LA. These regional differences may, for instance, be attributed to genetic factors previously associated with WMHs burden in FTD [56], highlighting the need for further investigation into potential mechanisms.

Our analysis also revealed comparable WMHs burdens in dementia groups from both geographic regions, with bvFTD showing a higher burden than AD. These results were independent of age, sex, and educational level and are consistent with findings from previous studies [9, 14]. To our knowledge, this is the first study examining WMHs burden in normal aging and dementia within a large LA cohort, extending findings observed in populations from the Global North to an underrepresented region. This study contributes to understanding the differences in aging and dementia phenotypes between the Global South and North [57], emphasizing the importance of including underrepresented populations where neurodegeneration and WMHs may exhibit stronger associations.

### Limitations and future directions

This study has several limitations that should be acknowledged. First, no biomarker data (such as PET imaging, CSF, or blood-based markers) were available to confirm the clinical diagnoses of dementia subtypes. Pathological confirmation remains the gold standard for diagnosis, and the significant overlap in clinical presentations, atrophy patterns, and even biomarker profiles across dementia subtypes further complicates definitive diagnosis. Notably, aside from the typical atrophied frontal areas observed in the bvFTD groups, the LA group also exhibited more posterior precuneal and cuneal atrophy compared to US participants. These differences may reflect underlying genetic factors, as the more widespread atrophy patterns observed with certain genetic mutations, including in the precuneal regions [58], contrast with the more focal patterns seen with other mutations [59]. Additionally, socio-environmental influences may impact the different atrophy patterns observed between LA and the US [60]. Further research incorporating genetic and environmental data would be necessary to validate these hypotheses. Second, while extensive cognitive testing was performed to diagnose dementia subtypes, the only cognitive measure consistently available across participants was the MMSE, with other cognitive tests having substantial missing data. This limited cognitive data restricts the ability to explore distinct clinical subtype presentations and their associations with WMHs. Future studies with more comprehensive cognitive assessments and biomarker data will be crucial for a deeper understanding of the neurobiological underpinnings of dementia subtypes.

Additionally, while this multisite study enhances brain research in LA, the inclusion of participants from multiple sites with varying scanning protocols may introduce uncontrolled variability, even after controlling for scanner effects. However, the LST toolbox used for WMHs mapping and volume extraction has shown to be reliable for multisite studies [61]. Despite this, larger and more balanced samples across regions are needed for systematic comparisons.

Furthermore, while comparing a more heterogeneous and vulnerable sample from LA with a seemingly more homogeneous and favorable one from the US, we may overlook a crucial aspect—the significant socioeconomic and ethnic heterogeneity within the US population. Despite this, our extensive literature review revealed a notable gap in WMHs studies, particularly regarding the representation of the diverse US population [23]. Consequently, we acknowledge that our US sample may not fully capture this heterogeneity, warranting caution when contrasting it with the LA sample.

The restricted accessibility to neuroimaging technology in some regions may also introduce selection bias, limiting generalizability. Exploring local factors, such as social determinants of health, will be critical to understanding regional differences in WMHs burden and cognitive performance, particularly in LA [62, 63]. Future research should incorporate cardiovascular and protein deposition biomarkers, genetic profiling, and protective factors (e.g., cognitive engagement, social interactions, prosocial behavior) to fully understand the interplay between WMHs burden, neurodegeneration, and cognition [64].

## Conclusion

This study highlights a more pronounced interaction between white and gray matter pathology within the LA population, as well as a greater burden of WMHs in HCs from LA compared to those from the US. This vulnerability, which affects key cognitive-related areas, may increase the risk of developing dementia. Our findings underscore the importance of investigating underrepresented populations to better understand regional differences in WMHs burden, paving the way for tailored interventions and global approaches to brain health.

## Supplementary Information

Below is the link to the electronic supplementary material.

Supplementary Material 1

## Data Availability

The data used in the analyses of this study are available. For ReDLat data, specific research projects can be submitted to the board for approval of a data-sharing agreement (https://red-lat.com). The code used in this study is available from the corresponding author upon reasonable request.

## Abbreviations

AD: Alzheimer’s Disease
AAL: Automated Anatomical Labeling
ANOVA: Analysis of Variance
bvFTD: Behavioral Variant Frontotemporal Dementia
CAT12: Computational Anatomy Toolbox, version 12
CSVD: Cerebral Small Vessel Disease
FLAIR: Fluid-Attenuated Inversion Recovery
FWE: Family-Wise Error
FWHM: Full Width at Half Maximum
GM: Grey Matter
HCs: Healthy Controls
LA: Latin America
LPA: Lesion Prediction Algorithm
LST: Lesion Segmentation Toolbox
MNI: Montreal Neurological Institute
MMSE: Mini-Mental State Examination
MRI: Magnetic Resonance Imaging
NINCDS-ARDA: National Institute of Neurological and Communicative Disorders and Stroke - Alzheimer’s Disease and Related Disorders Association
R²: Coefficient of Determination
SPM: Statistical Parametric Mapping
TIV: Total Intracranial Volume
US: United States
WM: White Matter
WMHs: White Matter Hyperintensities
