# Supplementary material for: White matter hyperintensities and their impact in brain structure and function in alzheimer’s disease and behavioral variant frontotemporal dementia across Latin America and the United States: a cross-sectional study
Source: Alzheimers Res Ther. 2025 Aug 12;17:188. doi: 10.1186/s13195-025-01832-5 (PMC12341313; doi:10.1186/s13195-025-01832-5)
Supplement: Supplementary file 1 — Supplementary Material 1 [file 13195_2025_1832_MOESM1_ESM.docx]

# Supplementary Material

All grey matter (GM) atrophy studies were done via voxel-based morphometry analysis using normalized and smoothed GM maps in Statistical Parametric Mapping software (SPM12,<https://www.fil.ion.ucl.ac.uk/spm/software/spm12/>). Two sample t-tests (SPM module) were run between groups (healthy controls (HCs), Alzheimer’s disease (AD), behavioral variant frontotemporal dementia (bvFTD)) and regions (Latin American countries (LA) and United States of America (US)), controlling for age, sex, total intracranial volume (TIV), and scanner. Results are family wise error-corrected for multiple comparisons at the voxel level (extended threshold = 50 voxels). Images are displayed in neurological convention.

## Supplementary Figure 1: Grey matter atrophy patterns in AD and bvFTD across LA and US


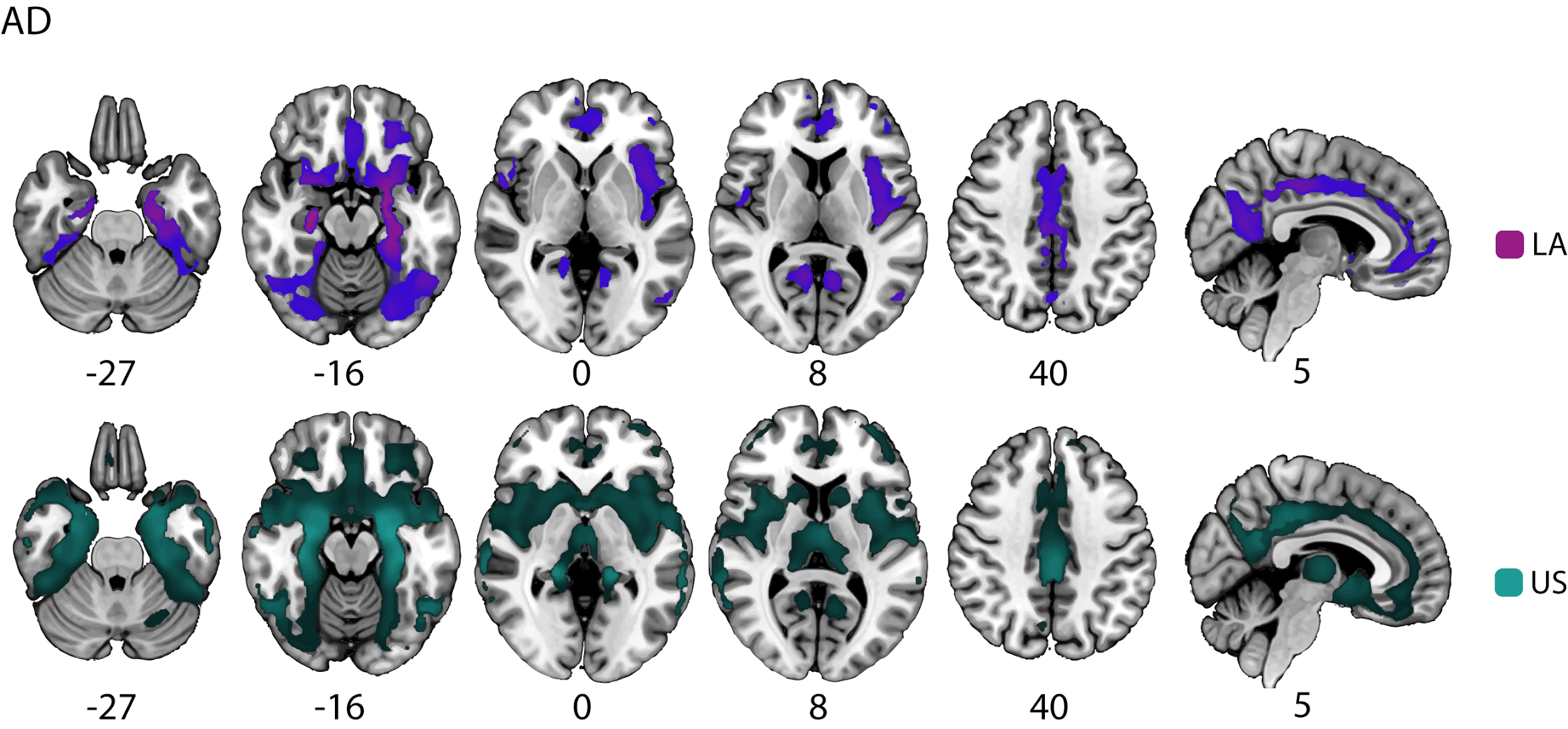

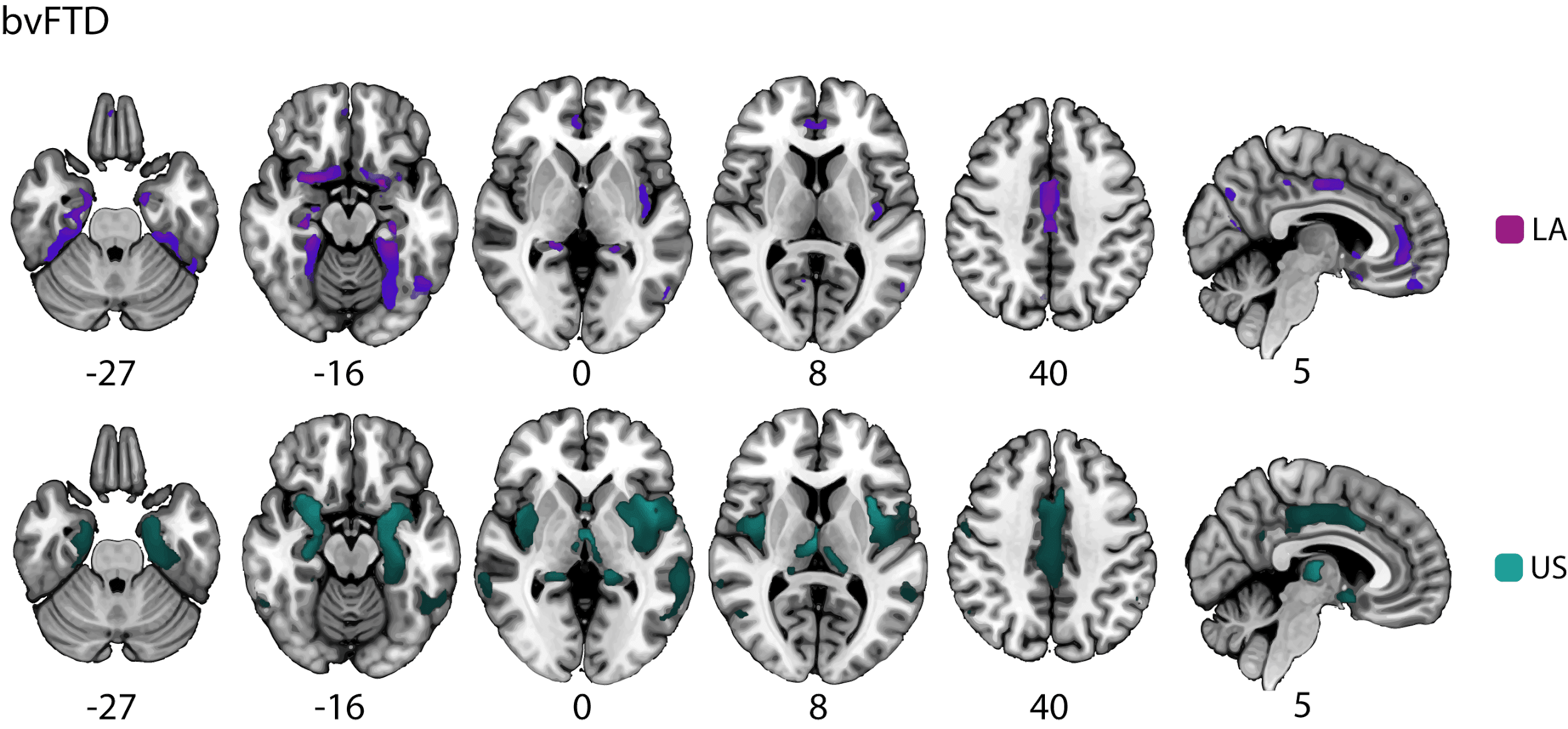


Brain maps showing GM atrophy patterns in dementia patients from LA and US. Top panel: AD participants from LA showed atrophy in the hippocampus, parahippocampal gyrus, superior and middle temporal gyrus, among other regions (Table S1). AD participants from the US showed atrophy in the hippocampus and the angular gyrus, among other regions (Table S2). Bottom panel: bvFTD participants from LA showed atrophy in the insula, anterior and medial cingulate cortex, hippocampus, amygdala and the medial orbitofrontal cortex, among other regions (Table S1). bvFTD participants from US showed atrophy in the insula, olfactory, middle cingulate cortex, precentral gyrus, and temporal areas, among others (Table S2). AD: Alzheimer’s disease, bvFTD: behavioral variant frontotemporal dementia. LA: Latin American countries. US: United States of America.

**Supplementary Table 1: Grey Matter atrophy areas in AD and bvFTD patients compared to HC in LA**

|  | **Brain Regions (AAL)** | **Cluster** | | **Peak** | **MNI Coordinates** | | |
| --- | --- | --- | --- | --- | --- | --- | --- |
|  |  | **N° voxels** | ***P* (FWE)** | ***t*** | **x** | **y** | **z** |
| **LA region** | | | | | | | |
| AD | Hippocampus R | 490 | 8,36E-04 | 12,96 | 27 | -13,5 | -16,5 |
|  | Hippocampus R |  |  | 12,64 | 28,5 | -22,5 | -13,5 |
|  | ParaHippocampal R |  |  | 11,86 | 18 | -9 | -18 |
|  | Hippocampus L | 25109 | 0,00E+00 | 12,75 | -25,5 | -10,5 | -16,5 |
|  | ParaHippocampal L |  |  | 12,53 | -22,5 | -37,5 | -3 |
|  | Hippocampus L |  |  | 12,41 | -28,5 | -31,5 | -10,5 |
|  | Amygdala R | 660 | 3,39E-04 | 11,13 | 19,5 | 0 | -13,5 |
|  | Temporal Pole Sup R |  |  | 10,89 | 31,5 | 7,5 | -21 |
|  | Insula R |  |  | 6,34 | 31,5 | 19,5 | -15 |
|  | Temporal Pole Sup R | 407 | 1,35E-03 | 7,99 | 51 | 4,5 | -1,5 |
|  | Rolandic Oper R |  |  | 6,76 | 49,5 | -10,5 | 9 |
|  | Rolandic Oper R |  |  | 6,48 | 52,5 | -4,5 | 4,5 |
|  | Temporal Mid R | 186 | 5,91E-03 | 7,77 | 52,5 | -63 | 22,5 |
|  | Frontal Mid L | 319 | 2,33E-03 | 7,76 | -34,5 | 39 | 31,5 |
|  | Frontal Mid L |  |  | 6,95 | -31,5 | 55,5 | 10,5 |
|  | Frontal Mid L |  |  | 6,63 | -27 | 51 | 21 |
|  | Frontal Mid L | 147 | 8,08E-03 | 6,36 | -43,5 | 37,5 | 15 |
|  | Frontal Inf Tri L |  |  | 4,98 | -43,5 | 45 | 3 |
| bvFTD | Olfactory L | 125 | 1,65E-02 | 9,52 | -22,5 | 3 | -16,5 |
|  | Hippocampus R | 2135 | 2,96E-05 | 9,35 | 31,5 | -24 | -13,5 |
|  | Hippocampus R |  |  | 8,03 | 27 | -12 | -18 |
|  | Fusiform R |  |  | 7,77 | 27 | -31,5 | -18 |
|  | Amygdala R | 318 | 6,29E-03 | 8,99 | 27 | 6 | -18 |
|  | Olfactory R |  |  | 6,54 | 10,5 | 13,5 | -18 |
|  | Hippocampus L | 3060 | 3,92E-06 | 8,57 | -28,5 | -28,5 | -12 |
|  | Hippocampus L |  |  | 7,99 | -18 | -9 | -18 |
|  | Fusiform L |  |  | 7,97 | -28,5 | -31,5 | -21 |
|  | Cingulum Mid L | 902 | 7,70E-04 | 7,77 | -3 | 7,5 | 39 |
|  | Cingulum Mid L |  |  | 7,60 | 0 | -10,5 | 43,5 |
|  | Cingulum Mid L |  |  | 7,09 | -3 | -24 | 43,5 |
|  | Frontal Med Orb L | 949 | 6,66E-04 | 7,56 | 0 | 42 | -9 |
|  | Cingulum Ant L |  |  | 7,49 | -4,5 | 31,5 | 22,5 |
|  | Caudate L |  |  | 7,44 | -3 | 9 | -9 |
|  | Insula L | 748 | 1,26E-03 | 7,40 | -30 | 7,5 | -15 |
|  | Temporal Sup L |  |  | 6,72 | -40,5 | -12 | -9 |
|  | Rolandic Oper L |  |  | 5,85 | -39 | -10,5 | 13,5 |
|  | Temporal Mid L | 119 | 1,71E-02 | 6,34 | -54 | -63 | 13,5 |
|  | Temporal Mid L |  |  | 6,09 | -55,5 | -64,5 | 3 |
|  | Occipital Inf L |  |  | 5,67 | -54 | -67,5 | -4,5 |
|  | Cuneus L | 213 | 1,03E-02 | 5,60 | -4,5 | -72 | 30 |
|  | Precuneus R |  |  | 5,06 | 6 | -70,5 | 34,5 |
|  | Cuneus L | 154 | 1,40E-02 | 5,21 | 0 | -69 | 22,5 |
|  | Calcarine R |  |  | 5,00 | 7,5 | -58,5 | 10,5 |
|  | Calcarine L |  |  | 4,85 | -3 | -66 | 15 |

Results are FWE-corrected for multiple comparisons at the voxel level (extended threshold = 50 voxels). AAL: Automated Anatomical Atlas. AD: Alzheimer’s disease, bvFTD: behavioral variant frontotemporal dementia, LA: Latin American countries. L: left, R: right.

**Supplementary Table 2: Grey Matter atrophy areas in AD and bvFTD patients compared to HC in the US**

|  | **Brain Regions (AAL)** | **Cluster** | | **Peak** | **MNI Coordinates** | | |
| --- | --- | --- | --- | --- | --- | --- | --- |
|  |  | **N° voxels** | ***P* (FWE)** | ***t*** | **x** | **y** | **z** |
| **US region** | | | | | | | |
| AD | Hippocampus L | 75907 | 0,00E+00 | 19,08 | -27 | -10,5 | -13,5 |
|  | Hippocampus L |  |  | 17,78 | -28,5 | -30 | -9 |
|  | Hippocampus R |  |  | 17,77 | 27 | -10,5 | -13,5 |
|  | Angular R | 53 | 1,75E-02 | 10,38 | 54 | -58,5 | 30 |
|  | Frontal Mid R | 254 | 2,45E-03 | 7,81 | 30 | 51 | 25,5 |
|  | Frontal Mid R |  |  | 6,61 | 31,5 | 43,5 | 30 |
|  | Frontal Mid R |  |  | 5,94 | 21 | 54 | 28,5 |
|  | Frontal Sup R | 404 | 8,15E-04 | 7,41 | 33 | 57 | 12 |
|  | Frontal Mid R |  |  | 6,99 | 46,5 | 36 | 18 |
|  | Frontal Mid R |  |  | 6,19 | 45 | 45 | 7,5 |
|  | Frontal Sup Orb R | 94 | 1,06E-02 | 6,17 | 28,5 | 60 | -3 |
|  | Frontal Inf Oper R | 56 | 1,68E-02 | 5,33 | 55,5 | 12 | 13,5 |
| bvFTD | Insula L | 17154 | 0,00E+00 | 13,91 | -40,5 | 12 | 3 |
|  | Thalamus L |  |  | 13,12 | -1,5 | -12 | 4,5 |
|  | Insula R |  |  | 12,21 | 36 | 21 | -4,5 |
|  | Cingulum Mid R | 3963 | 1,88E-08 | 9,99 | 6 | 18 | 39 |
|  | Cingulum Mid L |  |  | 9,82 | -4,5 | 21 | 36 |
|  | Cingulum Mid L |  |  | 7,24 | -1,5 | -6 | 45 |
|  | Olfactory L | 596 | 7,75E-04 | 9,97 | -1,5 | 9 | -10,5 |
|  | Olfactory L |  |  | 9,01 | -10,5 | 10,5 | -13,5 |
|  | Precentral L | 50 | 2,27E-02 | 7,83 | -49,5 | 3 | 42 |
|  | Temporal Mid R | 1392 | 3,20E-05 | 7,41 | 64,5 | -33 | -3 |
|  | Temporal Mid R |  |  | 6,04 | 63 | -39 | -10,5 |
|  | Temporal Mid R |  |  | 5,80 | 61,5 | -42 | 4,5 |
|  | Precentral R | 195 | 6,99E-03 | 5,84 | 55,5 | 4,5 | 36 |
|  | Precentral R |  |  | 5,65 | 54 | -6 | 40,5 |
|  | Parietal Inf L | 208 | 6,40E-03 | 5,33 | -49,5 | -45 | 49,5 |
|  | Parietal Inf L |  |  | 4,59 | -42 | -46,5 | 45 |
|  | Parietal Inf L |  |  | 4,40 | -55,5 | -52,5 | 40,5 |
|  | Angular R | 215 | 6,11E-03 | 5,20 | 49,5 | -60 | 45 |
|  | Cerebellum Crus2 L | 334 | 2,97E-03 | 5,12 | -19,5 | -78 | -52,5 |
|  | Temporal Sup L | 57 | 2,11E-02 | 4,66 | -51 | -33 | 15 |

Results are FWE-corrected for multiple comparisons at the voxel level (extended threshold = 50 voxels). AAL: Automated Anatomical Atlas. AD: Alzheimer’s disease, bvFTD: behavioral variant frontotemporal dementia, L: left, R: right. US: United States of America.

**Supplementary Table 3: Structural MRI acquisition parameters per center.**

|  |  | T1 | | | FLAIR | | |
| --- | --- | --- | --- | --- | --- | --- | --- |
| **Location**  **n participants** | **Scanner Tesla** | **N° slices** | **Matrix dimension** | **Voxel size (mm)** | **N° slices** | **Matrix dimension** | **Voxel size (mm)** |
| **AR Center A**  **n = 55** | Philips Ingenia 3T | 160 | 224x224 | 1x1x1 | 256 | 256x22 | 1x1x6 |
| **AR Center C**  **n = 19** | GE Signa HDxt 1.5T | 156 | 256x256 | 1x1x1 | 400 | 400x21 | 0.5x0.5x7 |
| **AR Center B**  **n = 94** | Phillips Intera 1.5T | 175 | 256x256 | 1x1x1 | 256 | 256x22 | 1x1x6 |
| **BR Center A**  **n = 17** | Philips Achieva 3T | 288 | 288x180 | 0.8x0.8x1 | 256 | 256x22 | 1x1x6 |
| **CH Center A**  **n = 31** | Philips Intera 1.5T | 256 | 175x256 | 1x1x1 | 256 | 256x22 | 1x1x6 |
| **CH Center B**  **n = 24** | Siemens Skyra 3T | 224 | 208x224 | 1x1x1 | 310 | 320x24 | 0.7x0.7x6.3 |
| **CH Center C**  **n = 49** | Siemens Skyra 3T | 192 | 256x256 | 1x1x1 | 310 | 320x24 | 0.7x0.7x6.3 |
| **CH Center D**  **n = 34** | GE Signa HDxt 1.5T | 512 | 240x512 | 1.2x1.2x1.2 | 180 | 256x20 | 0.9x0.9x6.6 |
| **CH Center E**  **n = 44** | Siemens Avanto 1.5T | 256 | 144x232 | 1x1x1 | 256 | 256x22 | 1x1x6 |
| **CO Center A**  **n = 138** | Philips Achieva 3T | 448 | 310x448 | 0.5x0.5x0.5 | 1008 | 1008x32 | 0.2x0.2x4.4 |
| **CO Center B**  **n = 27** | Philips Achieva 3T | 256 | 170x256 | 1x1x1 | 1024 | 1024x23 | 0.2x0.2x6 |
| **PE Center A**  **n = 59** | Siemens Aera 1.5T | 192 | 160x192 | 1.2x1.2x1.2 | 192 | 256x44 | 1x1x4 |
| **PE Center B**  **n = 18** | Siemens Verio 3T | 256 | 192x256 | 1x1x1 | 384 | 240x512 | 0.5x1x0.5 |
| **MX Center A**  **n = 30** | Siemens 3T | 256 | 160x256 | 1x1x1 | 310 | 320x30 | 0.7x0.7x5.2 |
| **US Center A**  **n = 108** | Siemens Prisma 3T | 160 | 240x256 | 1x1x1 | 176 | 256x256 | 1x1x1 |
| **US Center B**  **n = 145** | Siemens TrioTim 3T | 160 | 240x256 | 1x1x1 | 160 | 512x512 | 1x0.5x0.5 |
| **US Center C**  **n = 102** | Siemens TrioTim 3T | 160 | 240x256 | 1x1x1 | 160 | 256x256 | 1x1x1 |

AR: Argentina, BR: Brazil, CH: Chile, CO: Colombia, MX: Mexico, PE: Peru, TE: echo time, TR: repetition time, US: United States of America.

**Supplementary Table 4.** Tract-specific WMHs burden in LA

|  | **WM tracts (NatbrainLab atlas)** | **Cluster** | | **Peak** | **MNI Coordinates** | | |
| --- | --- | --- | --- | --- | --- | --- | --- |
|  |  | **N° voxels** | ***P* (FWE)** | ***t*** | **x** | **y** | **z** |
| **LA** | | | | | | | |
| AD >HCs | Optic Radiations L | 12278 | 2,72E-05 | 7,87 | -29 | -72 | 10 |
|  | Cingulum L |  |  | 4,80 | -19 | -59 | 27 |
|  | Inferior Occipito Fronal Fasciculus R | 7848 | 1,90E-04 | 7,24 | 32 | -67 | 6 |
|  |  |  |  | 5,75 | 26 | -83 | 6 |
|  | Internal Capsule R | 7076 | 2,77E-04 | 6,79 | 23 | 5 | 23 |
|  | Cortico Spinal R |  |  | 6,44 | 23 | -4 | 25 |
|  | Internal Capsule R |  |  | 6,29 | 16 | 4 | 12 |
|  | Corpus Callosum R | 3001 | 2,69E-03 | 6,79 | 4 | -35 | 17 |
|  | Cingulum R |  |  | 4,87 | 13 | -43 | 7 |
|  | Corpus Callosum R | 1047 | 1,18E-02 | 5,76 | 2 | 29 | 3 |
|  | Inferior Longitudinal Fasciculus R | 2494 | 3,78E-03 | 5,55 | 32 | 1 | -31 |
|  | Inferior Occipito Fronal Fasciculus L | 1251 | 9,85E-03 | 5,20 | -20 | 36 | -5 |
|  | Cortico Spinal L | 1038 | 1,19E-02 | 5,11 | -22 | -3 | 26 |
|  | Internal Capsule L | 811 | 1,48E-02 | 4,94 | -29 | -30 | 19 |
|  | Cortico Spinal L | 100 | 3,71E-02 | 4,84 | -16 | 1 | 12 |
| bvFTD >HCs | Fornix R | 130643 | 4,64E-12 | 8,10 | 32 | 1 | -33 |
|  | Corpus Callosum R |  |  | 7,62 | 2 | 30 | 4 |
|  | Inferior Longitudinal Fasciculus L |  |  | 5,23 | -33 | -71 | 11 |
|  | Corpus Callosum R | 2753 | 8,75E-03 | 5,50 | 24 | -74 | 19 |
|  |  | 765 | 2,39E-02 | 5,29 | -15 | 2 | -2 |
|  | Internal Capsule L |  |  | 5,08 | -17 | -1 | 9 |
|  |  | 245 | 3,54E-02 | 4,81 | 22 | -38 | 67 |
|  |  | 445 | 2,99E-02 | 4,77 | 17 | -50 | -28 |
|  |  | 350 | 3,23E-02 | 4,74 | 46 | -63 | 1 |
|  |  | 102 | 4,13E-02 | 4,68 | -11 | -88 | -14 |
|  | Inferior Occipito Frontal Fasciculus R | 1157 | 1,89E-02 | 4,65 | 29 | -62 | -4 |
|  | Inferior Occipito Frontal Fasciculus R | 205 | 3,68E-02 | 4,64 | 21 | -75 | -6 |
|  |  | 79 | 4,25E-02 | 4,55 | 31 | -52 | -14 |
|  |  | 125 | 4,01E-02 | 4,50 | 46 | -38 | -12 |
|  |  | 501 | 2,87E-02 | 4,50 | 5 | -76 | -35 |
|  |  | 235 | 3,58E-02 | 4,47 | 17 | -30 | -39 |
|  | Cortico Ponto Cerebellum L |  |  | 4,27 | 15 | -35 | -31 |
|  | Inferior Cerebellar Pedunculus L | 51 | 4,43E-02 | 4,44 | -6 | -53 | -21 |
|  |  | 57 | 4,39E-02 | 4,35 | 39 | -29 | 18 |
| bvFTD>AD | Cortico ponto Cerebellum Right | 1956 | 6,29E-04 | 5,647 | 22 | -17 | 17 |
|  | Cortico spinal Right |  |  | 4,937 | 24 | -29 | 14 |
|  | Corpus callosum Right | 2364 | 3,48E-04 | 5,282 | 12 | 35 | -1 |
|  | Corpus callosum Right |  |  | 5,051 | 11 | 29 | -11 |
|  | Cingulum Left | 1414 | 1,48E-03 | 4,966 | -10 | 35 | 10 |
|  | Corpus callosum left |  |  | 4,822 | -20 | 29 | 18 |
|  | - | 215 | 1,85E-02 | 4,763 | -36 | -4 | -39 |
|  | Corpus callosum Right | 51 | 3,42E-02 | 4,597 | 31 | -36 | -5 |
|  | Cingulum Left | 58 | 3,31E-02 | 4,486 | -1 | 23 | 16 |
|  | Cingulum Left | 52 | 3,41E-02 | 4,368 | -9 | 28 | -14 |

Results are FWE-corrected for multiple comparisons at the voxel level (extended threshold = 50 voxels). AD: Alzheimer’s disease, bvFTD: behavioral variant frontotemporal dementia, LA: Latin American countries. L: left, R: right.

**Supplementary Table 5.** Tract-specific WMHs burden in US

|  | **WM tracts (NatbrainLab atlas)** | **Cluster** | | **Peak** | **MNI Coordinates** | | |
| --- | --- | --- | --- | --- | --- | --- | --- |
|  |  | **N° voxels** | ***P* (FWE)** | ***t*** | **x** | **y** | **z** |
| **US** | | | | | | | |
| AD>HCs | Cortico Spinal R | 26334 | 2,21E-09 | 10,01 | 1 | -30 | 24 |
|  | Cingulum L |  |  | 8,03 | 2 | 18 | 19 |
|  |  | 3254 | 7,63E-04 | 7,69 | 11 | 6 | -3 |
|  | Inferior Longitudinal Fasciculus R | 6907 | 4,92E-05 | 6,32 | 35 | -62 | 0 |
|  | Corpus Callosum R |  |  | 6,20 | 33 | -64 | 11 |
|  | Inferior Longitudinal Fasciculus L | 4706 | 2,36E-04 | 5,95 | -33 | -68 | 5 |
|  | Cortico Spinal L | 3104 | 8,69E-04 | 5,73 | -23 | -12 | 31 |
|  | Internal Capsule L |  |  | 5,40 | -21 | 21 | 19 |
|  | Internal Capsule L |  |  | 4,89 | -20 | 8 | 28 |
|  |  | 5137 | 1,71E-04 | 5,73 | 26 | -7 | 31 |
|  | Internal Capsule R |  |  | 5,60 | 24 | 14 | 22 |
|  |  | 1105 | 6,60E-03 | 5,53 | -14 | 19 | -13 |
|  | Inferior Longitudinal Fasciculus R | 400 | 1,80E-02 | 5,10 | 44 | -41 | -9 |
|  | Arcuate Posterior Segment L | 425 | 1,72E-02 | 5,04 | -44 | -43 | -8 |
|  | Inferior Longitudinal Fasciculus L |  |  | 4,68 | -43 | -31 | -11 |
|  | Cingulum R | 163 | 2,85E-02 | 4,88 | 18 | -54 | 37 |
|  | Corpus Callosum L | 622 | 1,26E-02 | 4,83 | -21 | -86 | 7 |
| bvFTD>HCs | Anterior Commissure R | 352997 | 0,00E+00 | 12,53 | -14 | 20 | -15 |
|  |  |  |  | 11,19 | 17 | 8 | -3 |
|  | Inferior Longitudinal Fasciculus R | 3253 | 2,88E-05 | 5,80 | 30 | -83 | 11 |
|  | Corpus Callosum R |  |  | 5,12 | 24 | -78 | 24 |
|  | - | 236 | 1,39E-02 | 5,32 | 13 | -38 | 59 |
|  | Cortico Ponto Cerebellum L | 1200 | 1,09E-03 | 5,06 | 23 | -56 | -40 |
|  | - | 243 | 1,35E-02 | 4,74 | -24 | -51 | -44 |
| bvFTD>AD | Cingulum Right | 69212 | 5,96E-11 | 8,20 | -17 | 25 | -15 |
|  |  |  |  | 6,89 | 3 | 29 | -5 |
|  | Inferior Occipitofrontal Fasciculus Left | 6389 | 7,67E-04 | 6,23 | -32 | -38 | -4 |
|  | Internal Capsule Left |  |  | 5,77 | -28 | -37 | 6 |
|  | Optic radiations Left |  |  | 5,08 | -31 | -41 | 15 |
|  | Anterior Commissure Left | 5232 | 1,30E-03 | 6,00 | -32 | -4 | -24 |
|  | Uncinate Left |  |  | 5,53 | -33 | -1 | -15 |
|  | Cingulum Right | 508 | 2,33E-02 | 4,76 | 14 | 16 | 36 |
|  | Internal Capsule Right | 1858 | 8,08E-03 | 4,71 | 12 | -25 | -27 |
|  | Internal Capsule Right |  |  | 4,53 | 2 | -23 | -24 |
|  | - | 200 | 3,32E-02 | 4,53 | -36 | -21 | 11 |

Results are FWE-corrected for multiple comparisons at the voxel level (extended threshold = 50 voxels). AD: Alzheimer’s disease, bvFTD: behavioral variant frontotemporal dementia, L: left, R: right, US: United States of America.

**Supplementary Table 6. Areas of decreased grey matter volume associated to greater total WMHs volume in LA**

| **Group** | **Brain Regions (AAL)** | **Cluster** | | **Peak** | **MNI Coordinates** | | |
| --- | --- | --- | --- | --- | --- | --- | --- |
|  |  | **N° voxels** | ***P* (FWE)** | ***t*** | **x** | **y** | **z** |
| **LA region** | | | | | | | |
| HCs | Rectus R | 5890 | 6,22E-15 | 6,83 | 10,5 | 16,5 | -21 |
|  | Frontal Med Orb R |  |  | 6,82 | 1,5 | 43,5 | -7,5 |
|  | Frontal Inf Orb R |  |  | 6,60 | 36 | 24 | -18 |
|  | Frontal Inf Tri L | 298 | 8,77E-04 | 5,96 | -48 | 27 | 19,5 |
|  | Frontal Inf Tri L |  |  | 5,35 | -43,5 | 43,5 | 13,5 |
|  | Temporal Inf L | 2392 | 4,23E-09 | 5,96 | -48 | -37,5 | -24 |
|  | Fusiform L |  |  | 5,84 | -34,5 | -42 | -21 |
|  | Fusiform L |  |  | 5,84 | -24 | -39 | -12 |
|  | Frontal Inf Tri L | 230 | 1,67E-03 | 5,77 | -46,5 | 13,5 | 25,5 |
|  | Frontal Mid Orb R | 332 | 6,47E-04 | 5,76 | 39 | 60 | -3 |
|  | Frontal Mid Orb R |  |  | 4,62 | 36 | 43,5 | -15 |
|  | Frontal Mid Orb R |  |  | 4,62 | 27 | 63 | -12 |
|  | Frontal Inf Oper L | 987 | 6,08E-06 | 5,61 | -51 | 13,5 | 4,5 |
|  | Frontal Inf Orb L |  |  | 5,55 | -42 | 18 | -3 |
|  | Amygdala L |  |  | 4,99 | -28,5 | 4,5 | -18 |
|  | Cerebellum Crus1 R | 1128 | 2,62E-06 | 5,48 | 40,5 | -55,5 | -27 |
|  | Cerebellum Crus1 R |  |  | 5,21 | 43,5 | -46,5 | -30 |
|  | Fusiform R |  |  | 5,10 | 31,5 | -76,5 | -15 |
|  | Frontal Inf Tri R | 218 | 1,88E-03 | 5,21 | 48 | 40,5 | 13,5 |
|  | Frontal Mid R |  |  | 4,85 | 48 | 33 | 19,5 |
|  | Cingulum Mid R | 331 | 6,53E-04 | 5,02 | 1,5 | -21 | 43,5 |
|  | Cingulum Mid L |  |  | 4,49 | -3 | -10,5 | 40,5 |
|  | Cingulum Mid L |  |  | 4,49 | -3 | 0 | 42 |
|  | Fusiform R | 102 | 6,98E-03 | 4,96 | 22,5 | -37,5 | -16,5 |
|  | Frontal Mid R | 75 | 1,01E-02 | 4,87 | 36 | 46,5 | 25,5 |
|  | Fusiform R | 148 | 3,99E-03 | 4,66 | 27 | -4,5 | -40,5 |
| AD | Frontal Sup Orb R | 5932 | 2,06E-11 | 7,16 | 13,5 | 16,5 | -21 |
|  | Rectus L |  |  | 5,97 | 0 | 25,5 | -15 |
|  | Frontal Mid Orb L |  |  | 5,94 | -28,5 | 39 | -13,5 |
|  | Frontal Mid Orb R | 743 | 2,28E-04 | 6,79 | 36 | 43,5 | -13,5 |
|  | Frontal Sup Orb R |  |  | 6,14 | 12 | 63 | -15 |
|  | Frontal Mid Orb R |  |  | 5,70 | 31,5 | 54 | -13,5 |
|  | Rolandic Oper L | 1133 | 3,92E-05 | 5,90 | -45 | -13,5 | 12 |
|  | Rolandic Oper L |  |  | 5,68 | -36 | -24 | 15 |
|  | Temporal Sup L |  |  | 5,33 | -46,5 | -30 | 15 |
|  | Cingulum Mid R | 935 | 9,29E-05 | 5,80 | 7,5 | -34,5 | 42 |
|  | Precuneus R |  |  | 5,40 | 18 | -57 | 13,5 |
|  | Precuneus R |  |  | 5,20 | 6 | -52,5 | 24 |
|  | undefined | 312 | 2,46E-03 | 5,48 | -21 | -67,5 | -55,5 |
|  | Cingulum Mid L | 330 | 2,19E-03 | 5,41 | -10,5 | 22,5 | 31,5 |
|  | Cingulum Mid L |  |  | 4,83 | -7,5 | 7,5 | 39 |
|  | Cingulum Ant L |  |  | 4,79 | -10,5 | 31,5 | 27 |
|  | Fusiform R | 124 | 9,88E-03 | 5,39 | 21 | -37,5 | -15 |
|  | Temporal Inf L | 56 | 1,93E-02 | 5,30 | -58,5 | -13,5 | -28,5 |
|  | Cerebellum 8 R | 543 | 6,33E-04 | 5,27 | 21 | -69 | -55,5 |
|  | Cerebellum 8 R |  |  | 4,32 | 7,5 | -69 | -46,5 |
|  | Temporal Mid R | 182 | 6,13E-03 | 5,16 | 52,5 | -63 | 13,5 |
|  | Angular R |  |  | 4,51 | 51 | -63 | 27 |
|  | Frontal Inf Tri L | 684 | 3,05E-04 | 5,13 | -37,5 | 25,5 | 3 |
|  | Frontal Mid L |  |  | 5,12 | -40,5 | 52,5 | 4,5 |
|  | Frontal Inf Tri L |  |  | 4,93 | -45 | 42 | 10,5 |
|  | Temporal Inf R | 294 | 2,76E-03 | 5,08 | 51 | -63 | -9 |
|  | Temporal Inf R |  |  | 5,00 | 52,5 | -55,5 | -13,5 |
|  | Temporal Inf R |  |  | 4,92 | 57 | -52,5 | -7,5 |
|  | Frontal Sup Medial L | 74 | 1,59E-02 | 5,01 | -6 | 48 | 21 |
|  | Frontal Med Orb L | 131 | 9,30E-03 | 5,01 | -10,5 | 61,5 | -13,5 |
|  | Cerebellum 6 R | 192 | 5,68E-03 | 4,70 | 22,5 | -60 | -27 |
|  | Cingulum Ant R | 51 | 2,05E-02 | 4,66 | 7,5 | 48 | 16,5 |
|  | Cingulum Ant R |  |  | 4,38 | 7,5 | 42 | 25,5 |
|  | Cerebellum 6 L | 501 | 7,96E-04 | 4,63 | -19,5 | -64,5 | -16,5 |
|  | Fusiform L |  |  | 4,31 | -28,5 | -63 | -13,5 |
| bvFTD | Rolandic Oper L | 1481 | 1,59E-05 | 8,16 | -40,5 | -10,5 | 12 |
|  | Rolandic Oper L |  |  | 6,63 | -42 | -6 | 3 |
|  | Temporal Sup L |  |  | 5,98 | -42 | -7,5 | -7,5 |
|  | Fusiform R | 5744 | 1,12E-10 | 6,52 | 28,5 | -39 | -18 |
|  | Fusiform R |  |  | 6,49 | 25,5 | -57 | -13,5 |
|  | Cerebellum Crus2 R |  |  | 6,10 | 33 | -70,5 | -37,5 |
|  | Rolandic Oper R | 757 | 2,93E-04 | 6,41 | 42 | -1,5 | 9 |
|  | Insula R |  |  | 5,75 | 40,5 | -13,5 | 10,5 |
|  | Temporal Sup R |  |  | 5,37 | 43,5 | -4,5 | -10,5 |
|  | Rolandic Oper R | 64 | 1,88E-02 | 6,12 | 52,5 | -1,5 | 6 |
|  | Rolandic Oper R |  |  | 5,46 | 54 | 7,5 | 1,5 |
|  | Fusiform L | 1987 | 2,76E-06 | 6,10 | -28,5 | -42 | -15 |
|  | Lingual L |  |  | 5,47 | -15 | -60 | 0 |
|  | Calcarine L |  |  | 5,37 | -9 | -61,5 | 6 |
|  | Frontal Mid Orb R | 51 | 2,16E-02 | 5,93 | 24 | 63 | -10,5 |
|  | Cingulum Ant R | 616 | 5,68E-04 | 5,81 | 6 | 43,5 | 13,5 |
|  | Olfactory L |  |  | 5,56 | -3 | 13,5 | -12 |
|  | Frontal Med Orb R |  |  | 5,53 | 1,5 | 36 | -10,5 |
|  | Cingulum Mid R | 1021 | 9,36E-05 | 5,76 | 4,5 | 4,5 | 43,5 |
|  | Cingulum Mid R |  |  | 5,63 | 4,5 | -33 | 43,5 |
|  | Cingulum Mid L |  |  | 5,57 | -4,5 | -28,5 | 43,5 |
|  | Fusiform L | 671 | 4,36E-04 | 5,69 | -45 | -54 | -19,5 |
|  | Occipital Inf L |  |  | 5,61 | -51 | -61,5 | -15 |
|  | Temporal Inf L |  |  | 5,42 | -52,5 | -46,5 | -24 |
|  | undefined | 570 | 7,13E-04 | 5,42 | 18 | 6 | -13,5 |
|  | Caudate R |  |  | 4,70 | 10,5 | 16,5 | -9 |
|  | Calcarine R | 51 | 2,16E-02 | 4,86 | 10,5 | -58,5 | 10,5 |
|  | Cerebellum Crus1 L | 114 | 1,18E-02 | 4,68 | -22,5 | -76,5 | -28,5 |
|  | Cerebellum Crus1 L |  |  | 4,33 | -16,5 | -82,5 | -30 |
|  | Lingual R | 53 | 2,11E-02 | 4,63 | 13,5 | -52,5 | 3 |
|  | Calcarine R |  |  | 4,58 | 9 | -55,5 | 9 |

P-FWE<0.05, 50 voxels extended threshold, covariates: age, sex, years of education, VIT. Neurological convention. AD: Alzheimer’s disease. bvFTD: behavioral variant frontotemporal dementia. FWE: family-wise error rate. HCs: Healthy controls. LA: Latin American Countries. L: Left, R: Right.

**Supplementary Table 7. Areas of decreased grey matter volume associated to greater total WMHs volume in the US**

| **Group** | **Brain Regions (AAL)** | **Cluster** | | **Peak** | **MNI Coordinates** | | |
| --- | --- | --- | --- | --- | --- | --- | --- |
|  |  | **N° voxels** | ***P* (FWE)** | ***t*** | **x** | **y** | **z** |
| **US region** | | | | | | | |
| AD | Fusiform R | 799 | 8,00E-05 | 5,65 | 27 | -45 | -13,5 |
|  | Fusiform R |  |  | 5,42 | 33 | -37,5 | -19,5 |
|  | Fusiform R |  |  | 5,20 | 43,5 | -40,5 | -22,5 |
|  | Frontal Inf Tri L | 94 | 1,08E-02 | 5,45 | -45 | 19,5 | -1,5 |
|  | Occipital Inf L | 54 | 1,74E-02 | 5,13 | -49,5 | -72 | -7,5 |
|  | Occipital Inf L |  |  | 4,72 | -48 | -66 | -15 |
|  | Temporal Sup L | 86 | 1,18E-02 | 5,08 | -43,5 | -1,5 | -9 |
|  | Temporal Sup L |  |  | 4,71 | -46,5 | 0 | 0 |
|  | Temporal Mid R | 120 | 8,22E-03 | 4,95 | 58,5 | -21 | -7,5 |
|  | Temporal Sup L | 73 | 1,37E-02 | 4,89 | -61,5 | -15 | 9 |
|  | Temporal Sup L |  |  | 4,83 | -60 | -3 | 4,5 |
|  | Temporal Inf R | 82 | 1,24E-02 | 4,87 | 45 | 3 | -39 |

P-FWE<0.05, 50 voxels extended threshold, covariates: age, sex, years of education, VIT. Neurological convention. AD: Alzheimer’s disease, FWE: family-wise error rate. L: Left, R: Right, US: United States of America.

**Supplementary Table 8:  Tract-specific WMHs burden associated to cortical thinning in LA and US.**

|  | **Brain Regions (NatBrainLab)** | **Cluster** | | **Peak** | **MNI Coordinates** | | |
| --- | --- | --- | --- | --- | --- | --- | --- |
|  |  | **N° voxels** | ***P* (FWE)** | ***t*** | **x** | **y** | **z** |
| **LA region** | | | | | | | |
| HCs | Cingulum R | 92793 | 1,04E-10 | 8,499 | 5 | -29 | 31 |
|  |  |  | 1,09E-09 | 8,116 | -12 | -45 | 9 |
|  |  |  | 2,19E-09 | 8,001 | -3 | -32 | 33 |
|  | Cortico spinal L | 266 | 4,34E-04 | 5,729 | -16 | 4 | 4 |
|  | Fornix L | 187 | 1,25E-03 | 5,497 | 0 | -6 | 6 |
|  | Optic Radiations R | 362 | 8,74E-03 | 5,046 | 39 | -40 | 1 |
|  | Inferior Cerebellar Pedunculus R | 681 | 1,10E-02 | 4,990 | 0 | -63 | -34 |
|  |  |  | 1,81E-02 | 4,866 | 10 | -59 | -29 |
|  |  |  | 2,76E-02 | 4,759 | 13 | -51 | -27 |
|  | Cingulum R | 51 | 1,13E-02 | 4,984 | 19 | -50 | -9 |
|  | Cingulum L | 159 | 1,52E-02 | 4,910 | -25 | -48 | 38 |
| AD | Corpus Callosum R | 30232 | 4,76E-09 | 7,811 | 7 | -37 | 22 |
|  |  |  | 2,00E-07 | 7,169 | -6 | -41 | 26 |
|  |  |  | 3,61E-07 | 7,064 | 49 | -51 | 2 |
|  | Inferior Longitudinal Fasciculus L | 10552 | 4,22E-06 | 6,612 | -35 | -3 | -35 |
|  |  |  | 1,41E-04 | 5,920 | -49 | -9 | -14 |
|  |  |  | 2,82E-04 | 5,776 | -30 | -1 | -16 |
|  | Uncinate R | 1080 | 4,45E-05 | 6,155 | 20 | 17 | -14 |
|  |  |  | 6,49E-03 | 5,072 | 9 | 15 | -11 |
|  | Inferior Longitudinal Fasciculus R | 5439 | 5,09E-05 | 6,128 | 38 | 5 | -35 |
|  |  |  | 1,44E-03 | 5,421 | 47 | -7 | -28 |
|  |  |  | 5,53E-03 | 5,111 | 52 | 3 | -20 |
|  | Superior Cerebelar Pedunculus R | 2235 | 5,42E-04 | 5,636 | 3 | -27 | -11 |
|  | Cortico spinal L | 772 | 5,25E-03 | 5,123 | -14 | -13 | -9 |
|  |  |  | 1,07E-02 | 4,952 | -23 | -14 | 3 |
|  | Corpus Callosum L | 166 | 6,80E-03 | 5,061 | -10 | 35 | -13 |
| bvFTD | Corpus Callosum L | 3506 | 1,25E-06 | 7,071 | -3 | 26 | 5 |
|  |  |  | 9,28E-03 | 5,067 | -9 | 17 | -14 |
|  | Cortico spinal R | 31002 | 7,33E-06 | 6,706 | 26 | -27 | 16 |
|  |  |  | 8,57E-06 | 6,673 | 26 | -24 | 33 |
|  |  |  | 1,82E-05 | 6,513 | 25 | -14 | 23 |
|  | Cingulum L | 1307 | 1,40E-04 | 6,069 | -5 | 11 | 26 |
|  | Corpus Callosum L | 4356 | 1,97E-04 | 5,992 | -21 | 31 | 14 |
|  |  |  | 8,03E-04 | 5,669 | -20 | 14 | 27 |
|  | Cortico spinal L | 1715 | 4,29E-04 | 5,815 | -24 | -13 | 1 |
|  |  |  | 4,42E-04 | 5,808 | -17 | -7 | 1 |
|  |  |  | 5,69E-04 | 5,749 | -25 | -14 | 10 |
|  | Fornix L | 1175 | 1,41E-03 | 5,535 | -34 | -2 | -26 |
|  | Cingulum R | 177 | 2,19E-03 | 5,429 | 7 | -3 | 37 |
|  | Corpus Callosum R | 813 | 4,94E-03 | 5,228 | 19 | 34 | 5 |
|  |  |  | 6,08E-03 | 5,175 | 17 | 33 | -3 |
|  |  |  | 1,98E-02 | 4,867 | 20 | 24 | 21 |
|  | Inferior Longitudinal Fasciculus R | 478 | 9,30E-03 | 5,067 | 30 | 2 | -33 |
|  | Uncinate L | 276 | 1,04E-02 | 5,039 | -32 | 0 | -10 |
|  | Optic Radiations L | 304 | 1,15E-02 | 5,011 | -30 | -64 | 7 |
|  | Corpus Callosum L | 272 | 1,20E-02 | 5,001 | -13 | -40 | 9 |
|  | Corpus Callosum R | 55 | 3,20E-02 | 4,736 | 2 | -27 | 22 |
| **US region** | | | | | | | |
| HCs | Cingulum L | 787 | 6,09E-03 | 5,016 | 1 | -2 | 30 |
|  |  |  | 6,31E-03 | 5,007 | 1 | -12 | 32 |
|  |  |  | 2,29E-02 | 4,667 | 1 | 12 | 25 |
|  | Anterior Commissure R | 388 | 1,02E-02 | 4,884 | 20 | 2 | -4 |
| AD | Cingulum L | 10781 | 2,41E-04 | 6,126 | -13 | 38 | 7 |
|  |  |  | 2,92E-04 | 6,079 | 1 | 26 | -8 |
|  |  |  | 6,17E-04 | 5,894 | -4 | 12 | -5 |
|  | Inferior Occipito Frontal Fasciculus R | 4881 | 1,02E-03 | 5,767 | 33 | -8 | -9 |
|  |  |  | 3,77E-03 | 5,430 | 45 | -20 | -9 |
|  |  |  | 4,92E-03 | 5,359 | 1 | -7 | 0 |
|  | Cingulum L | 491 | 1,55E-03 | 5,661 | -14 | -49 | -7 |
|  | Inferior Longitudinal Fasciculus L | 158 | 1,16E-02 | 5,127 | -38 | -30 | -15 |
|  | Cingulum R | 147 | 1,62E-02 | 5,033 | 21 | -47 | -10 |
|  | Cingulum L | 255 | 1,76E-02 | 5,010 | -21 | -27 | -29 |
|  | Cingulum R | 206 | 1,95E-02 | 4,981 | 9 | 45 | 6 |
|  |  |  | 2,60E-02 | 4,900 | 21 | 49 | -3 |
|  |  |  | 3,46E-02 | 4,817 | 18 | 47 | 7 |
|  | Cortico spinal L | 72 | 2,71E-02 | 4,888 | -18 | -3 | 10 |
|  | Cortico spinal R | 79 | 3,49E-02 | 4,814 | 17 | 5 | 1 |
| bvFTD | Uncinate L | 3331 | 3,60E-04 | 6,222 | -32 | 3 | -23 |
|  |  | 942 | 1,36E-03 | 5,867 | -9 | 13 | -15 |
|  | Cingulum L | 321 | 8,08E-03 | 5,373 | -1 | 33 | -4 |
|  | Uncinate R | 2949 | 9,69E-03 | 5,321 | 37 | 7 | -23 |
|  |  |  | 1,03E-02 | 5,303 | 40 | -12 | -14 |
|  |  |  | 1,20E-02 | 5,260 | 43 | -4 | -15 |
|  |  |  | 2,93E-02 | 4,995 | 11 | 16 | -17 |
|  | Cingulum R | 87 | 2,03E-02 | 5,106 | 20 | -35 | -2 |
|  | Inferior Occipito Frontal F | 65 | 3,69E-02 | 4,925 | 37 | 0 | -10 |

Results are FWE-corrected for multiple comparisons at the voxel level (extended threshold = 50 voxels). AD: Alzheimer’s disease, bvFTD: behavioral variant frontotemporal dementia, Hcs: Healthy Control, LA: Latin American countries. L: left, R: right.
